# VINNA for neonates: Orientation independence through latent augmentations

**DOI:** 10.1162/imag_a_00180

**Published:** 2024-05-31

**Authors:** Leonie Henschel, David Kügler, Lilla Zöllei, Martin Reuter

**Affiliations:** German Center for Neurodegenerative Diseases (DZNE), Bonn, Germany; A.A. Martinos Center for Biomedical Imaging, Massachusetts General Hospital, Boston, MA, USA; Department of Radiology, Harvard Medical School, Boston, MA, USA

**Keywords:** computational neuroimaging, deep learning, structural MRI, artificial intelligence, high-resolution, newborn brain

## Abstract

A robust, fast, and accurate segmentation of neonatal brain images is highlydesired to better understand and detect changes during development and disease,specifically considering the rise in imaging studies for this cohort. Yet, thelimited availability of ground truth datasets, lack of standardized acquisitionprotocols, and wide variations of head positioning in the scanner posechallenges for method development. A few automated image analysis pipelinesexist for newborn brain Magnetic Resonance Image (MRI) segmentation, but theyoften rely on time-consuming non-linear spatial registration procedures andrequire resampling to a common resolution, subject to loss of information due tointerpolation and down-sampling. Without registration and image resampling,variations with respect to head positions and voxel resolutions have to beaddressed differently. In deep learning, external augmentations such asrotation, translation, and scaling are traditionally used to artificially expandthe representation of spatial variability, which subsequently increases both thetraining dataset size and robustness. However, these transformations in theimage space still require resampling, reducing accuracy specifically in thecontext of label interpolation. We recently introduced the concept ofresolution-independence with the Voxel-size Independent Neural Networkframework, VINN. Here, we extend this concept by additionally shifting allrigid-transforms into the network architecture with a four degree of freedom(4-DOF) transform module, enabling resolution-aware internal augmentations(VINNA) for deep learning. In this work, we show that VINNA (i) significantlyoutperforms state-of-the-art external augmentation approaches, (ii) effectivelyaddresses the head variations present specifically in newborn datasets, and(iii) retains high segmentation accuracy across a range of resolutions(0.5–1.0 mm). Furthermore, the 4-DOF transform module together withinternal augmentations is a powerful, general approach to implement spatialaugmentation without requiring image or label interpolation. The specificnetwork application to newborns will be made publicly available asVINNA4neonates.

## 1 Introduction

Collections of neonatal brain Magnetic Resonance Images (MRIs) are indispensable tounderstand brain development and to detect early signs of potential developmentaldisorders. One of the key tasks in MRI analysis is automated segmentation, thelabeling of anatomical regions of interest (ROIs) that can be used for quantitativemodeling of healthy development, for analyses in population studies, forunderstanding disease effects as well as a starting point for further neuroimagingtasks. The segmentation of infant MRIs is a challenging and non-trivial undertakingdue to the rapid non-linear changes during the postnatal brain growth period,elevated levels of head motion, limited availability of congruent datasets, varyingintensity profiles across scanners, protocols and modalities, as well as theinversion of gray-white contrast around the age of 5–9 months (Ajayi-Obe et al., 2000;Dubois et al., 2014;Gilmore et al.,2012;Prastawa et al., 2005). Inthis paper, we focus on a sub-group of the infantpopulation—newborns—and present a four-degree of freedom (4-DOF)transform module to address two core challenges within this cohort: non-uniformimage resolutions (scaling) and increased variability of head positions (rigidtransformations = rotation and translation) during image acquisition. The4-DOF transform module is directly integrated into the network architecture andaddresses the variability of head positions internally. As such, it expands andgeneralizes the distinguishing feature, resolution independence, of the recentlypublished Voxel-size Independent Neural Network (VINN) (Henschel et al., 2022) by rotation and translationtransformations. We refer to our new framework as VINN with “internalaugmentations” (VINNA).

In contrast to adults, newborn head positions in the scanner are far more diverse dueto the scanning conditions (asleep) and overall smaller anatomy. While padding isoften used to stabilize the child’s head and to occupy the space between headcoil and participant (e.g., foam cushions, pads, mats, pillows, or visco-elasticmatters) (Copeland et al., 2021), itsstandardization is difficult. This results in diverse head orientations within thescanner and potentially high variations among imaging studies.

In addition, there is no de-facto standard resolution for newborn imaging. In thecase of research protocols, when more time is available, MRIs are often acquired athigher resolutions to address the small size of brain structures and strongerpartial volume effects (Dubois et al., 2019,^2021^;Liuet al., 2019). However, the range of recorded resolutions across researchand clinical studies is relatively large and heterogeneous, ranging from 0.5 mm to3.0 mm in past and recent research studies (e.g., NIH-PD (Evans, 2006), BCP (Howellet al., 2019), Developing Human Connectome Project (dHCP) (Bastiani et al., 2019;Makropoulos et al., 2018), HBCD (Volkowet al., 2021)). Similarly, resolutions are not standardized acrossatlases (UNC 0-1-2 Infant atlases (Shi et al.,2011), Imperial Brain Development atlases (Gousias et al., 2012;Kuklisova-Murgasova et al., 2011;Makropoulos et al., 2016;Serag et al.,2012)), that are often used to guide the automated labelingalgorithms.

Traditional tools for newborn or infant segmentation predominantly interpolate imagesto a single chosen resolution and harmonize the spatial head position via atlasregistrations (Cherel et al., 2015;Dai et al., 2013;Makropoulos et al., 2018;Prieto et al., 2019;Shi et al.,2010;Zöllei et al., 2020).Resampling of images can, however, result in loss of information, especially in thecontext of high-resolution label maps. Furthermore, atlas-guided approaches areusually highly dependent on registration accuracy. For newborns, registration isparticularly challenging due to lower tissue contrast, specifically in the T1wscans. Errors in the process are hence common and improvement of the registration,for example, with spatio-temporal information, anatomical constraints, and surfacemodels, is an active field of research (Ahmad et al.,2019;Bozek et al., 2018;Dubois et al., 2021;Garcia et al., 2018;Kuklisova-Murgasova et al., 2011;Lebenberg et al., 2018;Makropoulos etal., 2014;Shi et al., 2010).

The explicit definition of spatial and intensity features can be avoided by usingConvolutional Neural Networks (CNNs). In fact, fast deep-learning methods forsemantic segmentation are becoming increasingly popular for infant segmentation(Bui et al., 2019;Dolz et al., 2020;Kumaret al., 2018;Moeskops et al.,2015;Nie et al., 2016;Qamar et al., 2020;Wang et al., 2022,^2023^;Zeng & Zheng, 2018;Zeng et al., 2023;Zhang et al., 2015). Applicability of deep-learningapproaches, however, is generally restricted to domains where sufficiently largetraining datasets exist. While there have been several initiatives to collect largerneuroimaging cohorts of newborns and infants in recent years (Bastiani et al., 2019;Howell et al., 2019;Makropoulos et al.,2018;Volkow et al., 2021), theirsize is still relatively small compared to equivalent cohorts in the adultpopulation. Additionally, accompanying manual labels are sparse, due to highannotation costs (time and money) and non-uniform labeling protocols, limiting thepool for supervised training options further. Considering the newborn cohort, thevariability in resolution and head positioning is likely underrepresented in thepublicly available datasets, questioning whether a network trained on the availablepairs of scans and labels can be robust enough without additional augmentation.

**Fig. 1.**
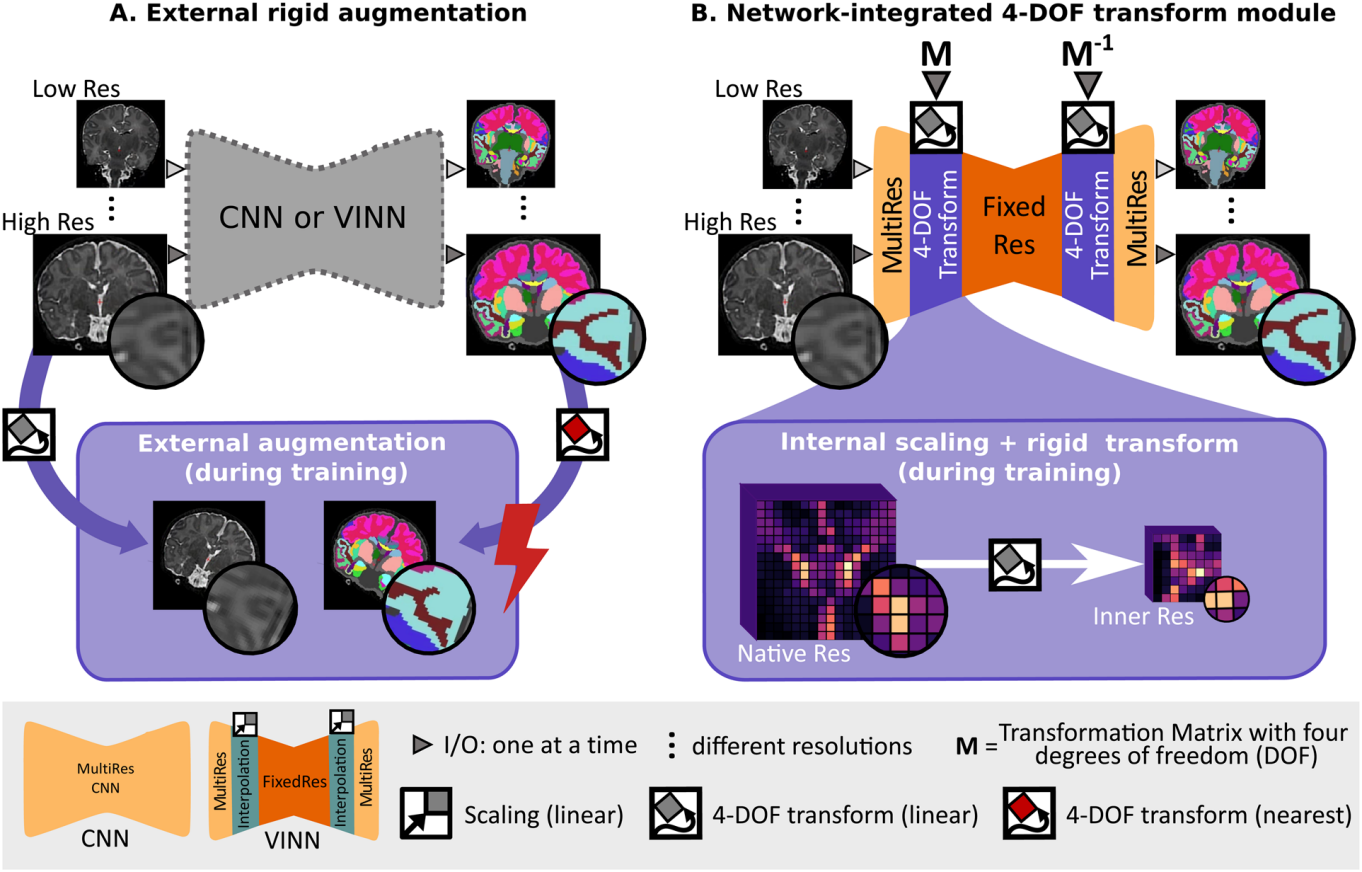
Spatial augmentations in deep-learning networks: (A) One singleresolution-ignorant CNN or resolution-independent voxel size independentnetwork (VINN) can learn to segment multiple resolutions and head positionsby training on a diverse dataset. External scale, rotation, and translationaugmentation (+ external augmentation, A. bottom) diversifies theexisting training samples by resampling the images and the referencesegmentation maps. Here, however, lossy interpolation and resultingartefacts, especially from nearest-neighbor interpolation of discrete labelmaps, may result in a loss of structural details and sub-optimalperformance. (B) Our 4-DOF transform module (VINNA) completely avoidsinterpolation of the images and discrete labels by integrating theinterpolation step into the network architecture itself (B. bottom).Rotations, translations, and scalings applied in the first interpolationblock are later reversed, assuring segmentations to be in the original imageorientation. Furthermore, the explicit transition from the native resolutionto a normalized internal resolution facilitates an understanding of thedifference between image features (MultiRes blocks with distances measuredin voxels) and anatomical features (FixedRes inner blocks with normalizeddistances).

The most widely used solution to artificially increase the training set size,robustness, and generalizability of deep-learning methods has been traditional dataaugmentation, such as rotation, scaling, or translation (Fig. 1A). In this case, both images and their labelmaps areinterpolated to a new random position during training. Interpolation, however, inthe native image space requires resampling of the discrete ground truthsegmentations, resulting in information loss (e.g., from lossy nearest-neighbor (NN)interpolation) and reduction in accuracy (Henschelet al., 2022).

With the VINN architecture (Henschel et al.,2022), we recently established the first network forresolution-independent deep learning, which effectively circumvents scalingaugmentation and subsequent external resampling, while leveraging information acrossdatasets of varying resolutions. In VINN, the classic fixed-factor integer down- andup-scale transitions, often implemented via pooling operations in UNet-likearchitectures (Ronneberger et al., 2015), arereplaced with a flexible re-scaling for the first and last scale transitions. Thisnetwork-integrated resolution-normalization allows for segmentation in the nativespace during both, training and inference. In adults, this approach has been shownto outperform fixed-resolution CNNs as well as resolution-ignorant CNNs trained withexternal scaling augmentation, and to improve performance both for sub-millimeterand one-millimeter scans.

Since newborn datasets are often acquired at various native resolutions and areparticularly subject to partial volume effects, the resolution-normalization featureoffers a basis to improve segmentation performance here as well. As is, VINN onlyaddresses scaling and would still require external augmentations, and hence labelinterpolation, to address the increased variability of head positions and limitedavailability of training data for newborns. With our VINNA and its 4-DOF transformmodule, we now close this gap and propose to shift away from classical dataaugmentation towards a detail-preserving internal augmentation scheme (Fig. 1B). While avoiding any type of labelinterpolation, we extend VINN’s network-integrated resolution-normalizationwith spatial augmentations (i.e., rotation and translation). At the first layerscale transition, the feature maps are hence not only flexibly rescaled, but alsorandomly transformed to imitate the position variations commonly found in newborns,subsequently increasing the training distribution.

In conclusion, VINNA and its 4-DOF transform module effectively address the challenges associated with newborn segmentation, namely variation in head positions and resolutions in the context of limited data availability. The four key contributions of VINNA presented in this work are as follows:

(i) We provide the first publicly availableopen-source deep-learning pipeline for a combined subcorticalsegmentation as well as cortical, and white matter parcellation fornewborn T1w or T2w MRIs.
(ii) We introduce a novel augmentation concept,which for the first time moves spatial augmentations into the network(instead of being performed outside). Our experimental results comparevarious spatial augmentation approaches side-by-side to isolate theireffects.
(iii) We ensure fair comparisons throughout, forexample, by fixed dataset splits, retraining methods under equal dataand parameter settings, comparing architectures and setups with minimaldifferences, and quantifying real-world performance.
(iv) We, further, provide extensive comparison withstate-of-the-art deep-learning methods (2D and 3D nnUNet) adapted fornewborn segmentation (retrained on the same data) and present anextensive comparison to the publicly available newborn segmentationpipelines iBEAT and infantFS.

The specific application of VINNA to newborns (approximately 24–44 weekspost-menstrual age) will be made available as VINNA4neonates within our open sourcerepository^1^including Dockercontainers offering easy accessibility for the community.

### 1.1 Related work

While various reliable and sensitive traditional (Fischl et al., 2002;Friston et al.,2007;Jenkinson et al., 2012;Zhang et al., 2001) and fastdeep-learning solutions exist (Billot et al.,2020;Chen et al., 2018;Coupé et al., 2020;Henschel et al., 2020,^2022^;Huo et al.,2019;Iglesias et al., 2021;Ito et al., 2019;McClure et al., 2019;Mehta et al., 2017;Roy et al.,2019;Sun et al., 2019;Wachinger et al., 2018) for adultwhole-brain segmentation, application of these methods to younger ages ishampered by the significant differences in size, MRI contrast profiles, andrapidly changing postnatal anatomy that is challenging to model with statictemplates.

### 1.2 Traditional tools for infant segmentation

Infant-specific traditional atlas-guided tools (Beare et al., 2016;Cherel et al.,2015;Makropoulos et al.,2018;Prieto et al., 2019;Shi et al., 2010;Zöllei et al., 2020) are predominantlyoptimized for a specific age range, resolution, and modality. Further, theydiffer significantly in the number of segmented classes and structuredefinitions.

The more recent Infant Brain Extraction and Analysis Toolbox (iBEAT) V2.0 (Wang et al., 2023) is a combination ofage-specific CNNs for tissue segmentation, traditional surface generation, andparcellation, based on atlas registration, into 34 regions following theDesikan-Killiany protocol (Desikan et al.,2006). It supports a large age range (0–6 years), and allowssegmentation of both, T1w and T2w MRI. While multiple input resolutions aresupported, iBEAT internally reorients and resamples each image to a standardformat (RAS orientation and 0.8 mm isotropic resolution). Hence, it does notsupport native resolution segmentation and image interpolation is required tomap segmentations back to the original input space. The resampling step isautomatically included in the pipeline such that in- and output resolutions areflexible. Furthermore, in its publicly available docker pipeline,^2^segmentation is limited to whitematter (WM), gray matter (GM), and cerebrospinal fluid (CSF).

infantFreeSurfer (infantFS) (Zöllei etal., 2020), on the other hand, mimics the FreeSurfer (Fischl, 2012) processing pipeline for adults andprocesses images from the first 2 years postnatally. It supports anatomicalsegmentation into 32 classes based on multi-atlas label fusion strategy,including registration to the infantFreeSurfer training data set (de Macedo Rodrigues et al., 2015). Theentire pipeline is publicly available^3^and allows processing of T1w images at a resolution of 1.0mm, where the atlas training data are defined. For newborns, T1w images oftensuffer from poor tissue contrast due to the underlying myelination process,aggravating accurate registration from the atlases onto individual brains. Thisage group can therefore be a challenge for infantFS’s mono-modalityapproach.

The dHCP minimal-processing-pipeline (Makropouloset al., 2018) is an optimized framework for cortical and sub-corticalvolume segmentation, cortical surface extraction, and cortical surfaceinflation, which has been specifically designed for high-resolution T2w MRIs ofnewborns (Hughes et al., 2017). Here, anExpectation-Maximization algorithm, including an atlas-based spatial prior term,labels 87 classes based on a modified version of the ALBERTs atlas (Gousias et al., 2012;Makropoulos et al., 2014). The segmentations includesubcortical structures, cortical and WM parcellations. Due to the cubic increasein voxel size for high-resolution images, processing times are in the order ofhours to days for a single subject. This is a common limitation amongtraditional methods.

### 1.3 Deep-learning for infant segmentation

#### 1.3.1 Newborns

Overall, networks for cortical parcellations and subcortical structuresegmentations in newborns are limited. The few existing CNNs support asingle modality (T2w), fixed resolution, and segment a single (Rajchl et al., 2017) or eight (Moeskops et al., 2015) tissue classes.One recent exception is the deep-learning based neuroimaging pipeline byShen et al. (2023), which istrained with the dHCP data. Here, the authors propose a 3D multi-task deeplearning model with a U-Net like architecture to segment structural T1w andT2w images on both thin and thick sliced images. Unfortunately, the networkfollows a fixed-resolution scheme (0.8 mm), it does not support nativesegmentation across resolutions commonly encountered in newborn cohorts, andit is not readily available online.

#### 1.3.2 Isointense phase

The vast majority of deep-learning tools focus on processing of images at theisointense phase around 6 months after birth (Ding et al., 2022;Dolz et al., 2020;Kumar et al., 2018;Nie et al., 2016;Pasban et al., 2020;Qamar etal., 2020;Zeng & Zheng,2018;Zeng et al., 2023;Zhang et al., 2015). Via theiSeg-challenge (Sun et al., 2021;Wang et al., 2019); data fortraining and validation are conveniently available, partly explaining thispredominance. While many interesting architectural solutions have arisen,the main focus of the works is the effective combination of information fromboth T1w and T2w images to generate a broad segmentation into CSF, GM, andWM. This modality combination is specifically important in the isointensephase, which is characterized by changes in the myelination stronglyeffecting the appearance of the recorded MRIs (Gui et al., 2012;Wang et al., 2015;Weisenfeld& Warfield, 2009). The inversion of the WM-GM signalresults in extremely low tissue contrast. The newborn cohort, on the otherhand, demonstrates good contrast between GM and WM, specifically on the T2wimages. While the age difference is small, the two cohorts as well as theassociated challenges are distinct and networks trained on the one cannoteasily be applied to the other. Subsequently, neitherresolution-independence nor the stronger variation of head positions isspecifically accounted for in network solutions for the isointensephase.

#### 1.3.3 Cross-age generalizability

To address generalizability across different age groups, recent research hassuggested optimized training strategies for neonates, such as multi-tasklearning of tissue segmentation and geodesic distances (Bui et al., 2019) or the use of inductive biasesin the form of pre-trained weights (i.e., fine-tuning to the target domain)(Wang et al., 2022). Bothapproaches improve segmentation accuracy, but they are still limited intheir generalizability. They require retraining and hence a sufficientamount of labeled data; additionally, they rely on private datasets,limiting their reproducibility. Recently, a contrast agnostic segmentationvia synthetic images, originally proposed for adult brain segmentation(Billot et al., 2020;Iglesias et al., 2021), has also beenadopted for infant segmentation (Shang etal., 2022). Unfortunately, the output resolution is fixed for thenetwork, and native resolution segmentations are not supported. Furthermore,while the model was able to generalize across a broader age range, thesynthetic images still differ considerably from real data and the network,therefore they underperformed compared to age-specific models trained onexisting MRIs. It should be noted that the authors did not aim to generaterealistic images but rather a better segmentation tool.

### 1.4 Resolution-independence and position transforms in deep learning

A general resolution-ignorant framework addressing position transforms viaexternal augmentations is nnUNet (Isensee etal., 2021). This network has successfully been applied for a varietyof segmentation tasks due to its inherent ability to construct optimal parametersettings based on the input data itself. It provides different network set-ups(2D, 3D, and a cascaded 3D approach for large images) as well as a number ofexternal image augmentations, including random rotation, scaling, mirroring, andgamma transformation. Interestingly, while the trained network also follows afixed-resolution scheme, pre- and post-processing automatically resamplesbetween original image and network resolution. While native resolutionsegmentation is not supported, in- and output resolutions are not fixed and themethod is therefore a valid alternative to our VINNA. Both, the 2D and 3D nnUNetwith external augmentation (exA) therefore serve as a state-of-the-art baselinefor the newborn segmentation task.

A siamese network for semi-supervised training of a network to become equivariantto elastic transformation has been proposed in a single-resolution setting(Bortsova et al., 2019). A dedicatedloss function assures that segmentations are consistent under a given class oftransformation applied first to the image, and second to the output. Theapproach therefore relies on external augmentation and applies thetransformation in the image space (before and after a UNet). The proposed VINNA,on the other hand, is fundamentally different. It shifts this step into thenetwork itself, hence creating an internal augmentation. Overall, the approachbyBortsova et al. (2019)does thereforeassure consistency across transformations in the labeling space, while VINNAtargets spatial consistency of the feature maps.

In spatial transformers (Jaderberg et al.,2015), transformations attempt to harmonize or re-orient the imageinto a better position. To this end, an affine transformation is implicitlylearned via a dedicated localisation network. Subsequent application of thecalculated coordinate grid resamples the source feature maps via bi-linearinterpolation to the new position. While our approach shares grid calculationand interpolation within the network with spatial transformers, our internalaugmentation approach is inherently different. First, spatial transformers donot diversify or augment feature maps, but rather try to reach a harmonizedposition with respect to the data seen during training. External augmentationsare still necessary to expose the network to a wide data variety and approximateequivariance. Second, instead of a localization network, we directly determinethe sampling-grid based on a transformation matrix, which allows for an explicitintegration of knowledge about the image, such as the resolution. As a result,computational complexity is reduced while achieving the desired positiondiversification and resolution-independence.

## 2 Material and Methods

### 2.1 Datasets

As the largest publicly available newborn dataset with intensity images,accompanying subcortical segmentations as well as cortical and WM parcellationsat the time of our experiments, we randomly assign participants from the dHCPcohort with corresponding T1w and T2w MRIs to the training, testing, andvalidation sets, while ensuring equal distribution of age and gender.Additionally, the Melbourne Children’s Regional Infant Brain (M-CRIB)atlas cohort serves as an independent testing set for external validation of thefinal method. Written informed consent to participate in this study was providedby the participants’ legal guardian or next of kin in accordance with theInstitutional Review Board. Complete ethic statements are available at therespective study webpages.

#### 2.1.1 dHCP

The developing Human Connectome Project (Makropoulos et al., 2018) includes T1w and T2w MRIs of newbornsimaged without sedation on a 3 T Philips Achieva scanner. It provides 0.5 mmisotropic de-faced scans of individuals imaged postnatally between 24 to 45weeks post-menstrual age. Imaging data for 578 participants with matchingT2w and T1w are selected. The original images were acquired in sagittal andaxial slice stacks with in-plane resolution of 0.8 mm × 0.8 mm and1.6 mm slices overlapped by 0.8 mm. Motion correction and super-resolutionreconstruction techniques (Cordero-Grande etal., 2018;Kuklisova-Murgasova etal., 2012) created isotropic volumes of resolution 0.5 mm. AllT1w scans follow the same inversion recovery multi-slice fast spin-echoprotocol with TR 4.795 s, TE 8.7 ms, TI 1.740 s, and SENSE factor 2.27(axial) / 2.56 (sagittal). The parameters for the T2w scans are TR 12 s, TE156 ms, and SENSE factor 2.11 (axial) / 2.66 (sagittal). The full dataset isavailable online.^4^In thepresent study, 318 quality-checked images are used for network training and90 for validation. A total of 170 images are used in the final test set.

Even though the dHCP follows a well-defined protocol, standardization ofpositioning during scanning is still a challenge. As shown inFigure 2, inter-subject head positiondiversity in the newborn cohort is larger than an equally standardized adultcohort (HCP).

**Fig. 2.**
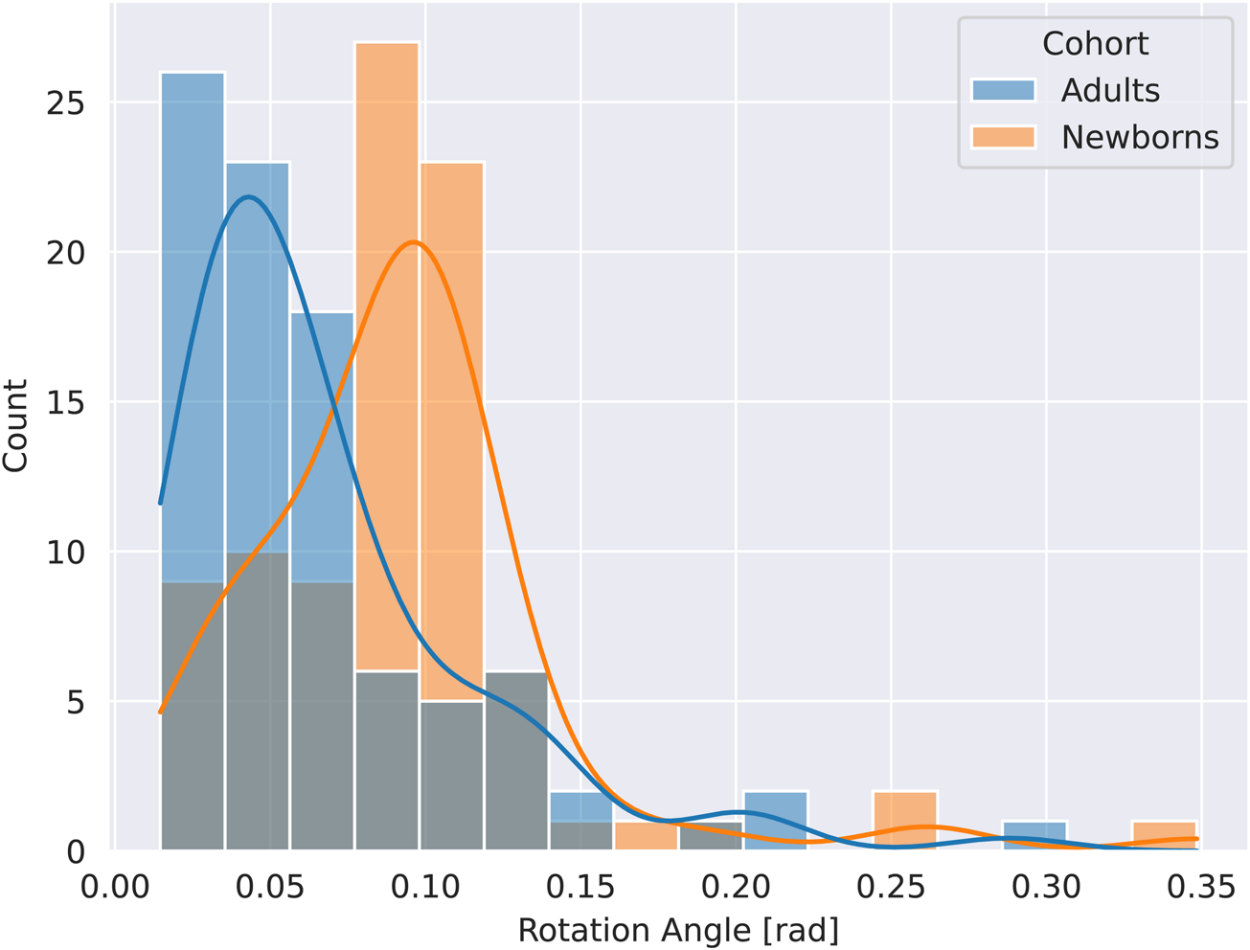
Comparison of variation in head position in newborns (orange) andadults (blue). Newborns show greater variation with respect torotation angles. Rotation transformation is based on alignment ofeach individual subject to their midspace. N = 90 for bothcohorts.

#### 2.1.2 M-CRIB

The Melbourne Children’s Regional Infant Brain (M-CRIB) atlas (Alexander et al., 2019) is constructedfrom 10 T2w MRI and corresponding manual segmentations of healthy term-bornneonates (four females, six males) with post-menstrual age-at-scan between40.29–43.00 weeks. The atlas comprises 94 neonatal brain regionscompatible with the widely-used Desikan-Killiany-Tourville adult corticalatlas (Klein & Tourville,2012). The T2w MRIs scanning protocols include the usage of atransverse T2 restore turbo spin echo sequence with 1.0 mm axial slices, aTR of 8.910 s, TE of 152 ms, flip angle of 120 degrees, Field Of View of 192mm × 192 mm, and in-plane resolution of 1 mm (zero-filledinterpolated to 0.5 mm × 0.5 mm × 1 mm). The T2w images arebias-corrected, skull-stripped, and resampled to 0.63 mm × 0.63 mm× 0.63 mm isotropic voxels. All 10 participants are used as anindependent testing set for our external validation experiments.

### 2.2 Generation of reference segmentation with the dHCP-minimal-processing-pipeline

To imitate various resolutions and create the desired reference segmentations fortraining, we processed all raw dHCP MRIs with thedHCP-minimal-processing-pipeline (Makropoulos etal., 2018) at 1.0 mm, 0.8 mm, and 0.5 mm. The structure definitionsfollow the ALBERTs atlas (Gousias et al.,2012) with the subdivision of the WM and cortex proposed byMakropolus et al. (2014), resulting in atotal of 87 structures (3 background labels, 20 subcortical regions, 32 corticalparcels, 32 WM parcels). We further lateralized the intracranial backgroundbased on the average Euclidean distance to neighboring labels, resulting in afinal count of 88 labels. We provide a list of all segmentation labels used fortraining in the Appendix (seeAppendix TableB). As the dHCP cohort includes both, T2w and a co-registered T1wMRIs, we trained dedicated networks for each modality. A manual quality checkfor all selected scans assured good overlap after the registration. Note thatthe dHCP-minimal-processing-pipeline relies on the original T2w images to createits segmentations, which are generally of higher quality in this collection.

### 2.3 Traditional infant segmentation tools

To evaluate VINNA against state-of-the-art traditional segmentation methods, wefurther process the testing set with the docker version of the iBEAT V2.0pipeline (Wang et al., 2023) and infantFS(Zöllei et al., 2020).

#### 2.3.1 iBEAT

The iBEAT V2.0 pipeline (Wang et al.,2023) combines both traditional and deep-learning models tocreate tissue segmentations into three classes (GM, WM, and CSF), surfacemodels, and cortical parcellations of the pial surface into 34 regions basedon the Desikan-Killiany protocol (Desikan etal., 2006). For tissue segmentation, iBEAT uses sevenage-specific CNNs trained on data for the representative age group(≤1, 3, 6, 9, 12, 18, and 24+ months of age). Neither thesource code nor the training data and labels are publicly available. Hence,retraining of the models is not possible and comparisons are limited to theiBEAT pipeline output as is. For processing with iBEAT, submissions via awebserver^5^orprocessing with a docker image^6^are possible. The docker version does not currentlysupport the cortical parcellations of the surface models. Due to the largenumber of participants, privacy concerns, and longer processing times whensubmitting via the webserver, we decided to use the docker version toprocess the T2w images of the testing set at the original 0.5 mm resolution.The resulting 3-label tissue segmentations form the basis for comparison tothe other tools in this paper.

#### 2.3.2 infantFS

To allow comparison of segmentation performance to VINNA, all available T1wimages from the dHCP testing set are processed with infantFS with defaultsettings. The neuroimaging pipeline infantFS creates surface models,anatomical segmentations, and cortical parcellations based on theDesikan-Killiany-Tourville atlas (Klein& Tourville, 2012) for 0- to 2-year-old infants akin tothe version for adults (FreeSurfer (Fischl,2012)). The tool runs on T1w MRIs at a resolution of 1.0 mm(non-conforming images are resampled). infantFS relies on aregistration-based multi-atlas label fusion strategy and returns ananatomical segmentation into 32 classes, including two labels for GM andWM.

#### 2.3.3 Label harmonization

**Fig. 3.**
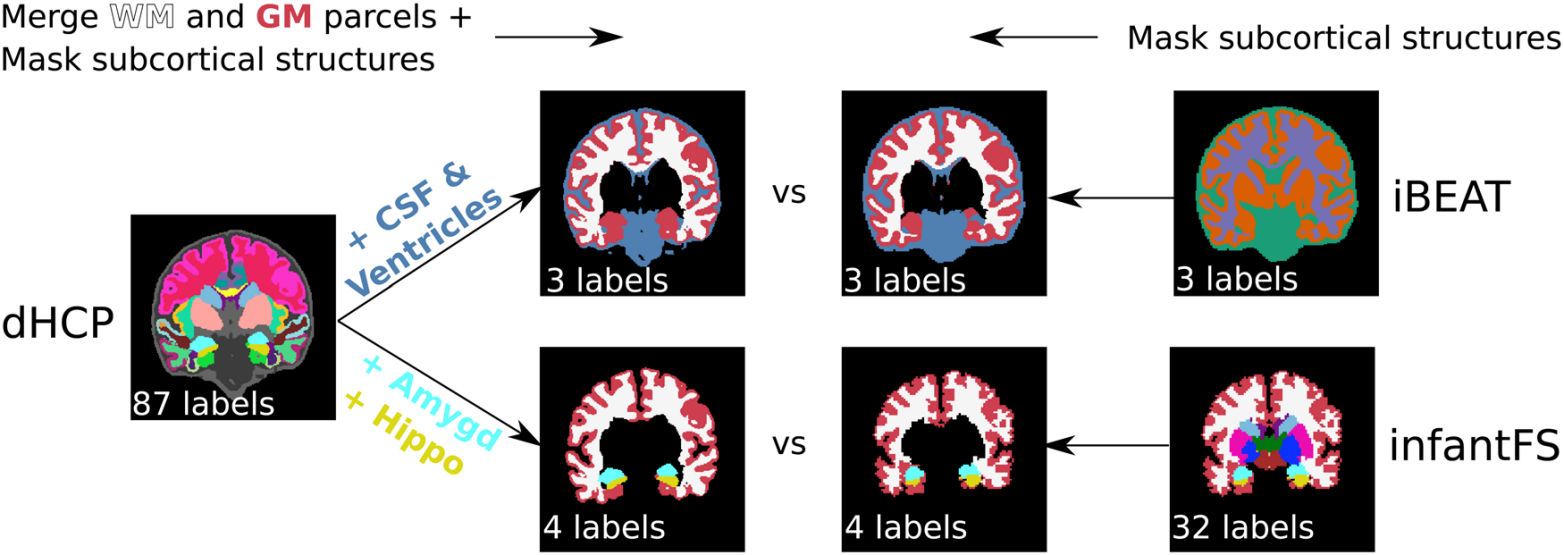
Harmonization of inconsistent label protocols between iBEAT,infantFS, and dHCP. Reduction of the original dHCP segmentation from88 labels (left) by first merging all cortical parcels to cortex, WMparcels to WM and removal of subcortical structures, is followed byaddition of CSF and ventricles (top) or amygdala and hippocampus(bottom) for comparison to iBEAT (3 labels: CSF (blue), WM (white),and GM (red), top second from left top) or infantFS (4 labels: WM(white), cortex (red), hippocampus (yellow), and amygdala (blue),bottom second from left), respectively. For iBEAT (right top), allGM and WM are modified using the dHCP segmentation for thesubcortical structures, resulting in three labels for the finalmapped version (3 labels, top second from right). For infantFS(right bottom), all structures except WM, cortex, hippocampus, andamygdala are removed (4 labels, bottom second from right).

As the dHCP-ALBERTs atlas differs from the resulting segmentations of bothiBEAT and infantFS, we merge, remove, and mask classes to reach anapproximate consensus across predictions.Figure 3shows the merging protocol on a representativeparticipant with the original and mapped ground truth dHCP labels (leftside) together with iBEAT (top right side) and infantFS (bottom right side).First, the 32 cortical and WM parcels from the dHCP ground truthsegmentation are reduced to two labels (cortex and WM) (top left inFig. 3). For iBEAT, the WM additionallyincludes the corpus callosum while GM also encompasses the hippocampus andamygdala. The CSF label corresponds to the union of lateral-ventricles andCSF in the dHCP-ALBERTs atlas. In the dHCP ground truth, these labels areconsequently merged to create the final three classes (GM, WM, and CSF; topsecond to left image). All other subcortical structures without a singlepossible assignment to GM, WM, or CSF are masked in the iBEAT predictionusing the dHCP ground truth (Fig. 3,top right two images). For infantFS, the hippocampus and amygdala labelremain, while individual cortex and WM parcels of the dHCP ground truth aremerged (Fig. 3, bottom left images).Hence, in the infantFS predictions the following four labels remain: cortex,WM, hippocampus, and amygdala (Fig. 3,bottom two images to the right).

### 2.4 Network architectures

#### 2.4.1 Macro architecture

**Fig. 4.**
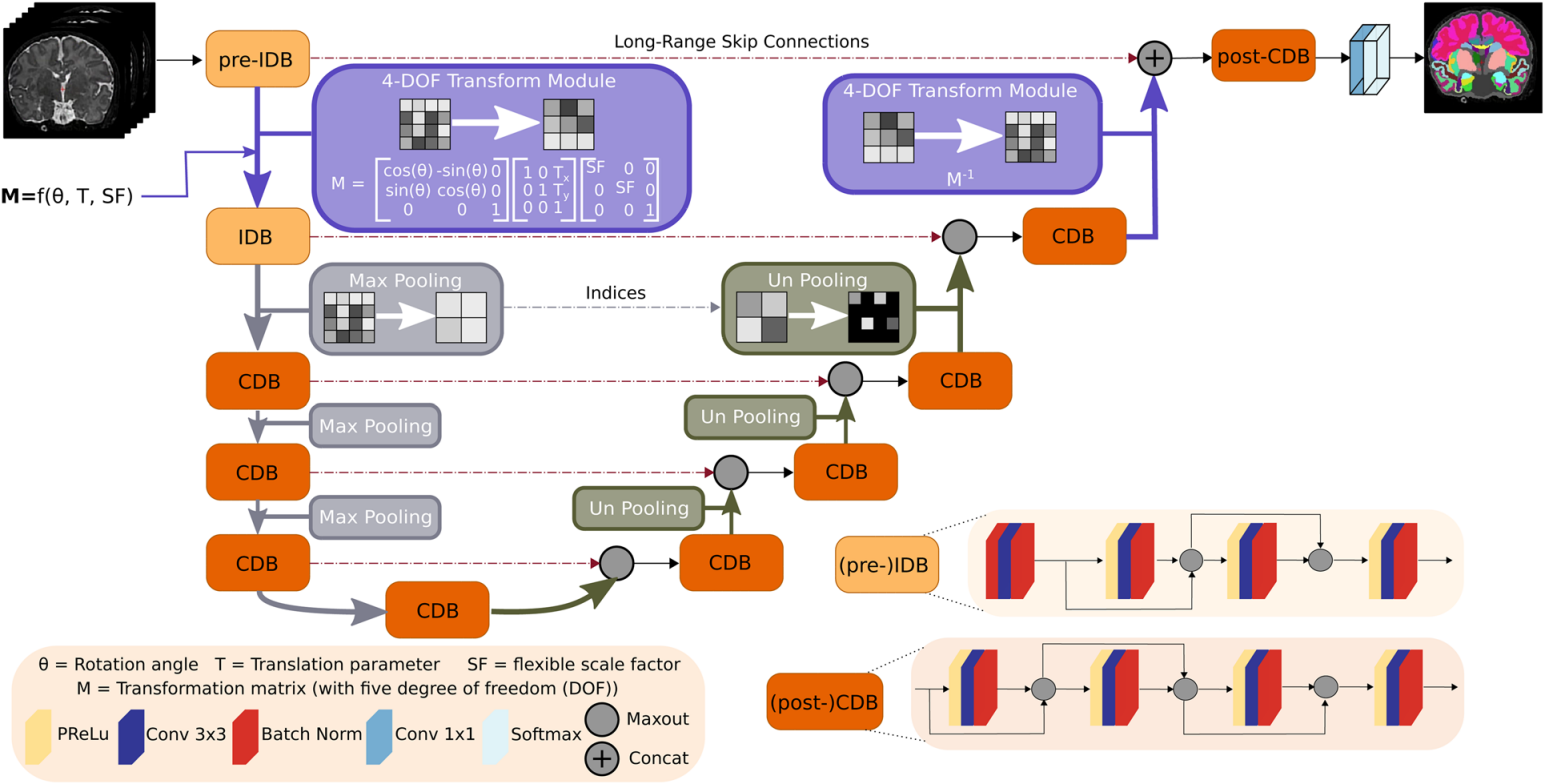
Network-integrated position variation and scaling normalization inVINNA. Flexible transitions between resolutions and head positionsbecome possible by replacing (un)pooling with our network-integrated4-DOF transform module (purple) after the first input dense block inthe encoder (pre-IDB) and before the last competitive dense block inthe decoder (post-CDB). A transformation matrix composed of rotationangleθ,translation parameter T, and the scaling factor SF defines thefeature alterations. Scale transitions between the other competitivedense blocks (CDB) remain standard MaxPool and UnPool operations.Each CDB is composed of four sequences of parametric rectifiedlinear unit (PReLU), convolution (Conv), and batch normalization(BN). In the first two encoder blocks ((pre)-IDB), the PReLU isreplaced with a BN to normalize the inputs.

Figure 4shows the macro architecturefor VINNA. While the proposed 4-DOF transform module (purple) can, intheory, be included in any UNet-like architecture, we use the same basicsetup for all trained models to assure maximum comparability (i.e., samenumber of parameters, same kernel sizes, etc.). The parameter-equal CNN isreferred to as CNN*. CNN*, VINN, and VINNA, all contain anencoder and decoder consisting of five competitive dense blocks,respectively, which are separated by a bottleneck layer. In the encoder,maxpooling operations rescale the feature maps at each level by one halfbetween the blocks using a pooling kernel of size2×2andstride 2. In contrast, index-unpooling doubles the feature map size in thedecoder. Skip connections between the blocks at each level allow thegradient to flow efficiently. In CNN* (Henschel et al., 2022), pooling and unpoolingoperations transition between all levels (i.e., the purple block inFigure 4is substituted with the graymaxpooling/unpooling operation). In VINN (Henschel et al., 2022), the first layer pooling and unpoolingoperation is replaced with a resolution-normalization module. Thisnetwork-integrated flexible interpolation step allows transitions betweenresolutions without restrictions to pre-defined fixed voxel sizes, bothduring training and inference. Hence, images can be processed at theirnative resolution without prior resampling. Similar to spatial transformers(Jaderberg et al., 2015), theinterpolation-based transition is divided into two parts: (i) calculation ofthe sampling coordinates (*grid generator*) and (ii)interpolation operation (*sampler*) to retrieve the spatiallytransferred output feature maps. Here, the sampling calculation relies onlyon the scale factor*SF*: the quotient of the resolutioninformation of the inner normalized scale Res_inner_, a tune-ablehyperparameter set to 0.8 mm throughout our experiments, and the input imageRes_native_. The addition of parameterαsampled from a Gaussian distribution with parameters sigma = 0.1 andmean = 0 slightly augments the scale factor SF ($SF={\text{Res}}_{\text{inner}}/{\text{Res}}_{\text{native}}+\alpha$),introduces small resolution variations to the sampling, and increases therobustness of the latent space interpolation. Specifically, the presence ofalpha allows for augmentations at the actual anatomical rather than thevoxel size as the normalization of the resolution inside VINN disentanglesperceived voxel versus actual structure size differences.

#### 2.4.2 Network-integrated 4-DOF transform module

In VINNA, the transition is implemented via the new network-integrated 4-DOFtransform module shown in purple inFigure4. Here, the sampling coordinate calculation is based on atransformation matrix$M\in {\text{R}}^{3\times 3}$with four degrees of freedom, encoding not only scaling, but also in-planerotation, and translation. Parameters for the rotation angleθ∈ℝand translation$T\;\in {\mathbb{R}}^{2}$are randomly sampled on the fly during training. The scale factor*SF*is calculated as in VINNs resolution-normalizationmodule, that is, by dividing the inner normalized scale by the imageresolution with augmentation by the parameterα.In the first transition step (Fig. 4,pre-IDB to IDB), the affine per-channel mappingM:U→Vsamples the inputfeature maps$U\in {\mathbb{R}}^{H_{\text{native}}\times W_{\text{native}}}$to the output feature maps$V\in {\mathbb{R}}^{H_{\text{inner}}\times W_{\text{inner}}}$.In the final transition step (Fig. 4,competitive dense block (CDB) to post-CDB), this spatial transformation isreversed by using the inverse transformation matrix$M^{-1}:V\to U$. The interpolationitself is performed by applying a sampling kernel to the input mapUto retrieve the value at a particular pixel in the output mapV.The sampling is identical for each channel, hence conserving spatialconsistency.

#### 2.4.3 Network blocks

##### 2.4.3.1 Competitive dense block (CDB) design

In VINNA, a CDB is formed by repetitions of the basic composite functionconsisting of a probabilistic rectified linear unit (pReLU) activationfunction, a3×3convolution, and a batch-normalization (BN). Feature competition withinthe block is achieved by using maxout (Goodfellow et al., 2013) instead of concatenations (Jégou et al., 2017) in thelocal skip connections. The maxout operation requires normalized inputsand is therefore always performed after the BN (see position of maxoutin CDB design inFig. 4).

##### 2.4.3.2 Input competitive dense block (IDB) design

In contrast to the described CDB, the first two network blocks follow adifferent order of operation. Here, the raw inputs are normalized byfirst passing them through a BN-Conv-BN combination before adhering tothe original composite function scheme (Conv-BN-pReLU) (seeFig. 4, IDB).

##### 2.4.3.3 Pre-IDB

The first encoder block in VINNA called pre-IDB (seeFig. 4) transfers image intensity information fromthe native image to the latent space and encodes voxel size andsubject-space-dependent information before the internal interpolationstep. The composite function scheme is identical to the IDB, and theadded prefix simply allows differentiation of the block placements.

##### 2.4.3.4 Post-CDB

Akin to the pre-IDB, an additional CDB block in the decoder merges thenon-interpolated feature information returned from the pre-IDB skipconnection and the upsampled feature maps from the network-integrated4-DOF transform modules. A concatenation operation combines both featuremaps, before passing them to a standard CDB block (seeFig. 4, (post-)CDB). After the final1×1convolution, a softmax operation returns the desired classprobabilities.

### 2.5 Loss function

All networks are trained with a weighted composite loss function of logistic lossand Dice loss (Roy et al., 2017) combinedwith the high-resolution specific weighting from VINN (Henschel et al., 2022). In short, erosion anddilation of the cortex labels creates a binary mask of the outer cortex, smallWM strands, and deep sulci. Wrong predictions in these areas result in a higherloss, hence guiding the network to focus on areas particularly affected bypartial volume effect (PVE). The original publications’ ablationexperiments evaluated the impact of the different function elements: thelogistic loss and Dice loss combination improves overall segmentationperformance (Roy et al., 2017), while thehigh-resolution weighting leads to higher segmentation accuracy on the corticalparcels (Henschel et al., 2022).

If we consider$p_{l,i}\left(x\right)$as the estimated probability of pixelithat belongs to classl,​y, as thecorresponding ground truth probability, and$\omega_{i}$as the associated weight given to the pixelibased the loss function can be formulated as

$$ℒ=\underset{\text{Logistic loss}}{\underset{︸}{-\sum_{l,i}\omega_{i}y_{l,i}\text{log}\;p_{l,i}\left(x\right)}}-\underset{\text{Soft Dice loss}}{\underset{︸}{\sum_{l}\frac{2\sum_{i}\;p_{l,i}\left(x\right)y_{l,i}}{\sum_{i}\;p_{l,i}\left(x\right)+\sum_{i}y_{l,i}}}}$$ (1)

with$\omega_{i}=\omega_{\text{medianfreq.}}+\omega_{\text{gradient}}+\omega_{\text{GM}}+\omega_{\text{WM/Sulci}}$.

Here,$\omega_{\text{medianfreq}}$.represents median frequency balancing addressing the class imbalance and$\omega_{\text{gradient}}$boundary refinement through a 2D gradient vector (Roy et al., 2017), while$\omega_{\text{GM}}$and$\omega_{\text{WM/Sulci}}$assign higher weights to PVE-affected areas (Henschel et al., 2022).

### 2.6 View aggregation

In order to account for the inherent 3D geometry of the brain, we adopt the 2.5Dview aggregation scheme from (Henschel et al.,2020,^2022^) for CNN*,VINN, and VINNA. In short, we train one network instance per anatomical planeand calculate a weighted average of the resulting softmax probability maps. Theweight of the sagittal predictions is reduced by one half compared to the othertwo views to account for the missing lateralization in the sagittal view. Inthis plane, the network predicts 23 instead of 88 classes.

### 2.7 Augmentations

#### 2.7.1 External augmentation (exA)

The current state-of-the-art approach to introduce robustness to positionchanges into neural networks is extensive external augmentation (seeFig. 1B). Therefore, we contrast ourproposed network-integrated 4-DOF transform module against this approach. Weuse random transforms with rotation parameters sampled from a uniformdistribution of the predefined range -180° to 180° andtranslation by 0 to 15 px to augment images during the training phase andinterpolate linearly. For CNN* and nnUNet, augmentation also includessampling of scaling parameters from a uniform distribution of the predefinedrange 0.8 to 1.15. VINN’s resolution-normalization module makes thisstep obsolete. Every minibatch hence consists of a potentially transformedMRI (using bi-linear interpolation) and a corresponding label map (using NNsampling). By exposing a network to a large variety of possible imagepositions during training, orientation-robustness can be achieved. Allexternal augmentation routines are implemented using torchIO (Pérez-García et al.,2021).

#### 2.7.2 Image intensity augmentation

To allow generalization outside of the dHCP cohort, we apply a number ofintensity or texture augmentations on the fly to the training batch, namelybias field changes, random gamma alterations, ghosting, spiking, blurring,and Gaussian noise. Each batch sampled from the original data is transformedby any of the operations above with a probability of 0.4. As before, allaugmentations are implemented using torchIO.

### 2.8 Evaluation metrics

We use the Dice Similarity Coefficient (DSC) (Dice, 1945;Sørensen,1948) and Average Surface Distance (ASD) to compare different networkarchitectures and modifications against each other, and to estimate similarityof the predictions with a number of previously unseen scans. Both are standardmetrics to evaluate segmentation performance. We establish improvements bystatistical testing (Wilcoxon signed-rank test (Wilcoxon, 1945) after Benjamini-Hochberg correction (Benjamini & Hochberg, 1995) for multipletesting) referred to as “corrected p” throughout the paper.

### 2.9 Training setup for all models

#### 2.9.1 Training dataset

For training, we select 318 representative participants from the dHCP cohort.Resolutions are equally represented with 106 MRIs at 1.0 mm, 0.8 mm, and 0.5mm, respectively. Empty slices are filtered from the volumes, leaving onaverage 137 single view planes per subject and a total training size of atleast 20k images per network. The original nnUNet does not filter thevolumes. The 3D version uses 3D patches instead of 2D slices. The parametersare automatically determined by nnUNet to guarantee an ideal set-up for thegiven segmentation task. Otherwise, we train all directly compared networks(CNN*, VINN, VINNA, 2D nnUNet, and 3D nnUNet) under the sameconditions.

#### 2.9.2 Training parameters

We implement and train independent models to convergence (early stopping) forthe coronal, axial, and sagittal planes with PyTorch (Paszke et al., 2017), using one NVIDIA V100 GPUwith 32GB RAM. During training, the modified Adam optimizer (Loshchilov & Hutter, 2019) is used with alearning rate of 0.001. Using a cosine annealing schedule (Loshchilov & Hutter, 2017) with warmrestarts, the learning rate is adapted after initially 10 epochs. The epochoffset is subsequently increased by a factor of two. The momentum parameteris fixed at 0.95 to compensate for the relatively small mini batch size of16 images for CNN*, VINN, and VINNA. For nnUNet, the optimalbatch-size is automatically determined (Isensee et al., 2021). For the given segmentation problem, the2D nnUNet uses a batch size of 128 while the 3D version relies on a smallerbatch size of 128. To ensure a fair comparison, all networks (CNN*,VINN, VINNA, 2D nnUNet, and 3D nnUNet) have been trained under equalhardware and hyper-parameter settings otherwise. A comparing table isavailable in the Appendix (SectionA.4).

## 3 Results

We group the presentation of results into three blocks: 1. ablative architectureimprovements to determine the best performing module for orientation and positiontransformation (Section 3.1), 2. performanceanalysis to comprehensively characterize the advantages of VINNA with respect tostate-of-the-art traditional atlas- and deep-learning-based methods (Section 3.2), and 3. external validation onM-CRIB (Alexander et al., 2019) to assesgeneralizability and performance with respect to manual labels (Section 3.3). Following best practice in data-science, weutilize completely separate datasets during the evaluations: the validation set forSection 3.1(Appendix Table A: Validation), and various test sets forSections 3.2and3.3(Appendix Table A: Testing).This avoids data leakage and ensures that training, method design decisions, andfinal testing do not influence each other, which could otherwise lead to overlyoptimistic results (overfitting).

### 3.1 External augmentation versus network-integrated 4-DOF transform module in VINNA

As high variances with respect to head orientations and spatial resolutions arecommon in newborns and are likely to be underrepresented in the limitedavailable data cohorts, we first compare multiple approaches for extension ofthe training distribution for accurate (sub)millimeter newborn whole-brainsegmentation. Traditionally, external data augmentation (exA), such as scaling,rotation, or translation, addresses this problem by interpolating both, theimage and label maps, to a new, random position during training. Due to thediscrete nature of the label maps, lossy NN interpolation cannot be avoided,unless the transformations are applied to the one-hot-encoded logits(soft-loss). We therefore evaluate both, the traditional exA and soft-lossimplementation (referred to as exA (soft)). Our new 4-DOF transform module inVINNA internally emulates possible head transformations and acts directly on theencoded feature maps. To evaluate effectiveness of the exA versus VINNA, wecompare VINNA with parameter-identical CNN*, VINN, and VINNA equippedwith exA. Each subsequent improvement in segmentation performance is confirmedby statistical testing (correctedp<0.05).

InFigure 5, we compare the modelperformance of six approaches: CNN* with exA and exA (soft), (Section 2.4, CNN* + exA, leftbox; CNN* + exA (soft), second box from the left), VINN with exAand exA (soft) (Section 2.4, VINN +exA, third box from the left; VINN + exA (soft), fourth box from theleft), VINNA with the new 4-DOF transform module (VINNA, second box from theright), and finally VINNA with exA (VINNA + exA, right box). The analysisof the DSC (top) and ASD (bottom) is grouped for three groups of structures(cortex averages 32 labels, WM averages 32 labels, and subcortical structuresaverage 20 labels) and three resolutions (from left to right 0.5 mm, 0.8 mm, and1.0 mm). We present performance for T2w MRIs, but we found the same ranking forT1w MRIs.^7^

**Fig. 5.**
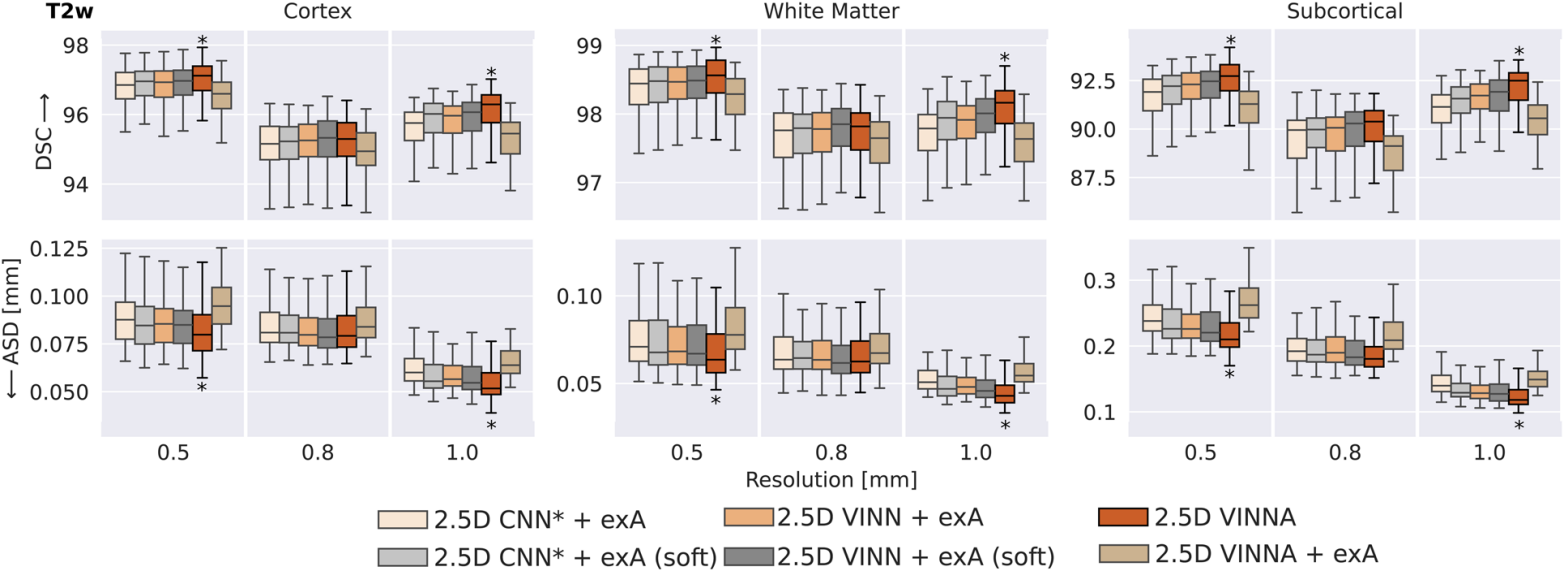
Comparison of approaches for external and internal spatial augmentationon T2w images: Our VINNA method—with the 4-DOF transformmodule—(fifth box in the group) outperforms both state-of-the-artapproaches with external Augmentation (CNN* + exA and VINN+ exA, first and third box) in Dice Similarity Coefficient (DSC,upper row) and average surface distance (ASD, lower row). Thisperformance advantage is significant (corrected$p<10^{-9}$)and consistent across resolutions and structures. Combining VINNA withexternal augmentation (VINNA + exA, last box) reducesperformance. When avoiding nearest-neighbor label interpolation byapplying exA on one-hot encoded logits (+exA (soft)) instead ofthe label maps, performance improves for both CNN* and VINN(second box and fourth box, respectively). VINNA with the internal 4-DOFmodule outperforms the soft-loss methods on 1.0 and 0.5 mm by asignificant margin (corrected$p<10^{-9}$,indicated with*). exA: external Augmentation.

Looking at the T2w segmentation and focusing on the different resolutions, thedifferences between the approaches are largest on the subcortical structures.The CNN* with exA reaches an average DSC of 91.91, 89.95, and 91.14 andan ASD of 0.238 mm, 0.192 mm, and 0.193 mm for input data of 0.5 mm, 0.8 mm, and1.0 mm, respectively. The slight reduction in performance for 0.8 mm resolutionconsistently occurs for all evaluated models and is probably caused by thenecessary image resampling from the original resolution of 0.5 mm and subsequentreprocessing with the dHCP-pipeline (Section2.2). Interpolation from 0.5 mm to 1.0 mm results in a well-alignedgrid due to the even division by factor 2 (8 voxels get averaged into a singlelarger voxel). Resampling to 0.8 mm, on the other hand, requires an uneveninterpolation grid with weighted averages and original voxels that contribute tomultiple larger voxels. This more challenging setting could result in theslightly reduced segmentation performance. Optimization of the architecturedesign towards multi-resolution (VINN,Section2.4) leads to significant improvement in the DSC and ASD across thecortical, WM, and subcortical structures (Fig.5, VINN + exA). Particularly, the subcortical segmentationsare improved by around 0.5%. Importantly, the internal 4-DOF transform module(VINNA), which avoids label interpolation all together, further reduces theerror by one half and increases segmentation performance significantly comparedto both CNN* and VINN with exA. This effect is consistent across allstructures and resolutions. Specifically, the ASD at the high-resolutionbenefits from the new module. Here, performance can be improved by 4.47% on thecortex, 5.19% on the WM, and 5.89% on the subcortical structures. For the lowerresolution, the improvement on the cortex and WM is slightly lower (around 2%)while the subcortical structures benefit from the 4-DOF transform modulesimilarly to the 0.5 mm resolution experiments.

The soft-loss, implementing augmentations for the label maps via linearinterpolation of the one-hot encoded feature maps, does improve performancecompared to the nearest-neighbor based external augmentation for bothCNN* and VINN (see CNN*/VINN + exA versus CNN*/VINN+ exA (soft)). The subcortical structures improve the most (0.27% DSC and5.1% ASD for CNN* + exA versus + exA (soft); 0.21% and2.35% ASD for VINN + exA versus + exA (soft)), followed by thecortex (0.15% DSC and 3.7% ASD; 0.07% DSC and 1.8% ASD). The improvedperformance without nearest-neighbor interpolation further strengthens ourrecommendation to avoid this type of label interpolation wherever possible. Theproposed VINNA architecture outperforms VINN + exA (soft) on the 0.5 and1.0 mm resolution by 0.17% and 0.35% DSC and 5.27% and 6.23% ASD, respectively.The subcortical structures benefit the most with an average improvement by 0.34%DSC and 4.39% ASD, respectively. On the 0.8 mm resolution, no significantdifference is detectable between the two approaches.

Overall, VINNA reaches the highest DSC and lowest ASD for the cortical structures(96.24, 0.079 mm), WM (98.18, 0.063 mm), and subcortical structures (91.87,0.190 mm) across all resolutions. The addition of exA to the framework (VINNA+ exA, right box in each plot) again reduces performance. The DSC dropsby 0.4%, 0.28%, and 1.15% on the cortex, WM and subcortical structures onaverage, while the ASD worsens by 4.75%, 3.92%, and 4.84%. Overall, results withVINNA are significantly better on 0.5 mm and 1.0 mm compared to all ablations onthe validation set (corrected$p<10^{-6}$).

### 3.2 Comparison to state-of-the-art neonate segmentation tools

To evaluate how VINNA compares to state-of-the-art neonate MRI segmentationtools, namely nnUNet (3D and 2D), iBEAT, and infantFS, we take a closer look atthe DSC and ASD on the testing sets.

#### 3.2.1 Comparison of deep-learning networks

**Fig. 6.**
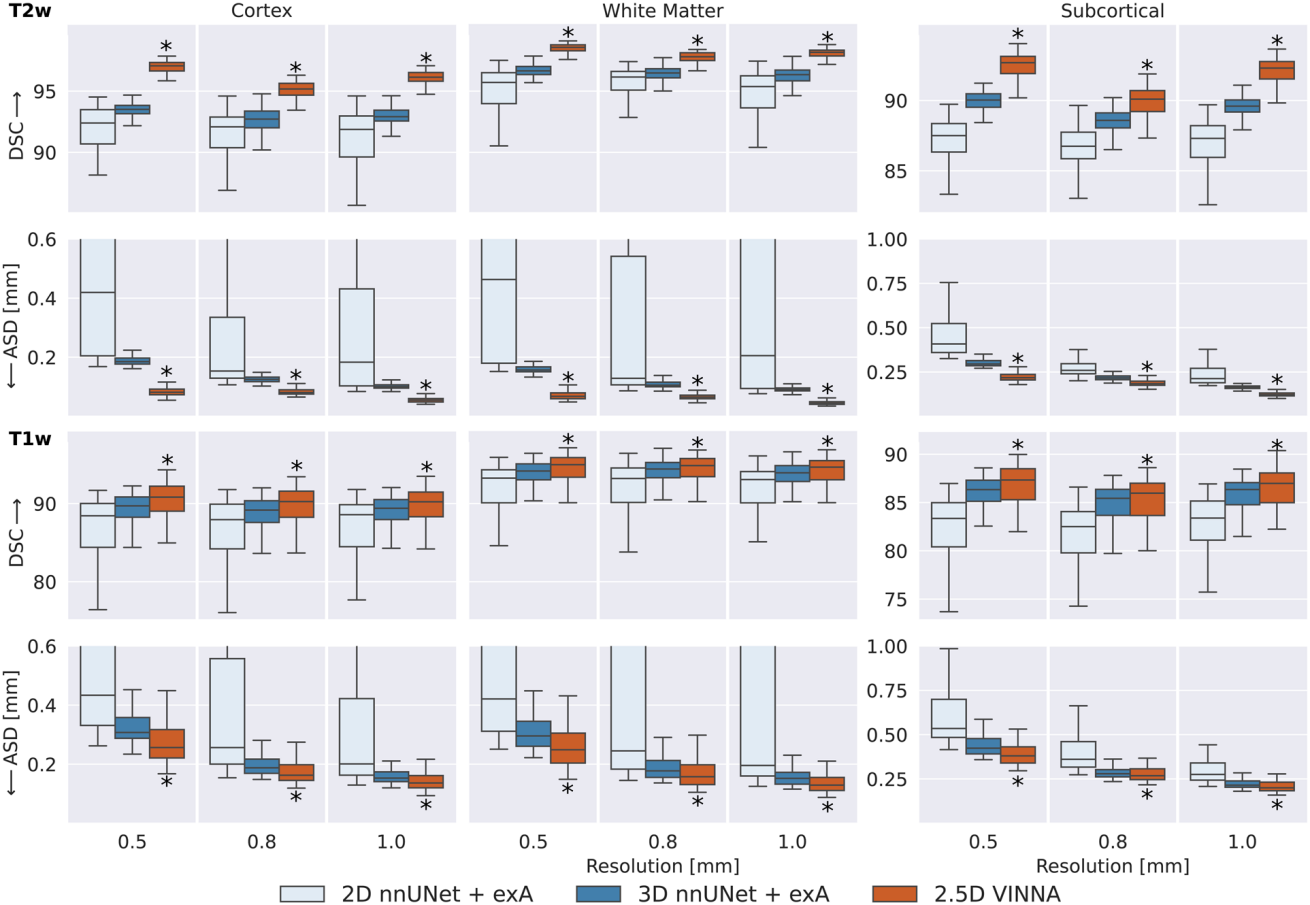
SOTA Segmentation performance: Our VINNA with the 4-DOF transformmodule (last box in group) outperforms the three state-of-the-artdeep-learning approaches, 2D nnUNet + exA and 3D (first andsecond box) in Dice Similarity Coefficient (DSC, upper row) andaverage surface distance (ASD, lower row). This performanceadvantage is significant (corrected$p<10^{-10}$,indicated with *) and consistent across three resolutions(0.5 mm, 0.8 mm, and 1.0 mm), two modalities (T2w, top and T1w,bottom), and three structure groups (cortex, WM, and subcorticalstructures).

Figure 6shows a detailed comparison ofthree different deep-learning based methods for neonate segmentation acrossmodalities (T2w top, T1w bottom) and resolutions. All models are trainedunder equal parameter and dataset settings. Comparing performance betweenthe two modalities shows that all models perform better on the T2w (top)than the T1w MRIs (bottom) across all structures. With VINNA (right box ineach plot), the reduction is similar across all resolutions, with an averagedifference in DSC of 5.85, 3.47, and 5.16 on the cortex, WM, and subcorticalstructures. The ASD is, on average, improved by 0.09 mm when predicting onthe T2w instead of T1w inputs. The nnUNet framework in 2D (left box) and 3D(second from left) has less improvement on the T2w images with an averagedifference between T1w and T2w of 3.75, 2.44, and 3.70 in DSC and 0.04 mmASD on the aforementioned structures.

When comparing the four models, the 2D nnUNet + exA (left box) versionperforms worse than the 3D (second from left box), and 2.5D VINNA (rightbox) across all resolutions, structures, and modalities. Particularlynotable are the large variations of 2D nnUNet + exA in predictionperformance (large standard deviation) and large ASD (seeFig. 6), especially at the highestresolution (0.42 mm, for the cortex, 0.46 mm for WM and 0.41 mm forsubcortical structures). This difference is less prominent in the DSCscores, but 2D nnUNet + exA also performs worst across allresolutions with respect to this metric (i.e., 92.39, 95.70, and 87.50 for aresolution of 0.5 mm). The 3D nnUNet + exA (second from left)improves accuracy by 24.5%, 12.3%, and 22.9% for ASD and 1.75%, 1.06%, and1.64% for DSC across the three different resolutions. VINNA with its 4-DOFtransform module (VINNA, right box) is again the best performing model,significantly outperforming all other networks. Compared to the 3D nnUNet+ exA, ASD and DSC scores are significantly improved with the highestgain on the cortical structures (56%, 35%, and 45% ASD and 3.8%, 2.7%, and3.5% DSC for 0.5 mm, 0.8 mm, and 1.0 mm, respectively).

**Fig. 7.**
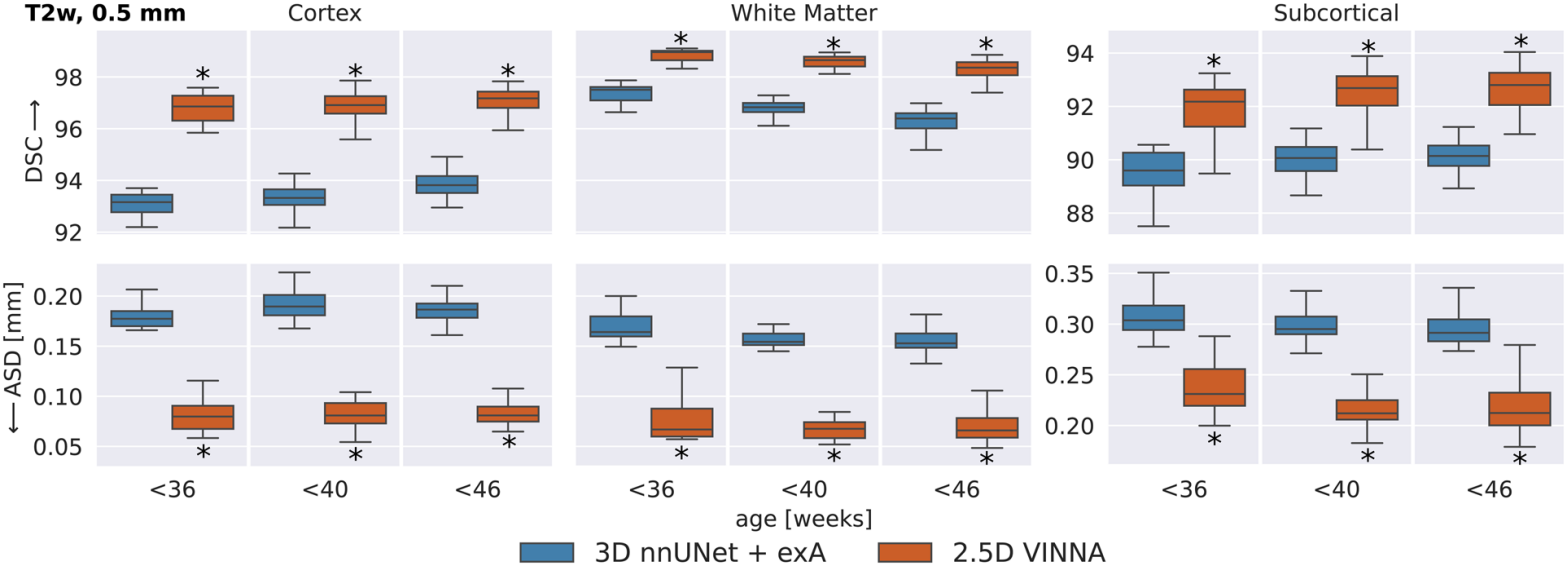
SOTA Segmentation performance across age groups: VINNA equipped withthe 4-DOF transform module (right box) consistently outperformsstate-of-the-art deep-learning approaches 3D nnUNet with externalaugmentation (+exA, left box) across four age groups.Improvement in dice similarity coefficient (DSC, top) and averagesurface distance (ASD, bottom) on T2w at 0.5 mm is significant(corrected$p<10^{-4}$,indicated with *) for<36,<40,and<46week-old newborns.

To evaluate if this trend is consistent across age groups, the 0.5 mm MRIimages are split into three approximately equal-sized groups based on theparticipants’ age-at-scan information (32–36, 36–40,and 40–46 weeks).Figure 6showsDSC (top) and ASD (bottom) calculated for T2w (Fig. 7) for the 3D nnUNet + exA (left box) and VINNA withits 4-DOF transform module (right box) in each of the categories.

Consistent with the previous section, 3D nnUNet + exA reaches theweakest ASD and DSC across all age groups. On average, the VINNA with its4-DOF transform module (right box) improves performance compared to nnUNetby 2.8%, 2.9%, and 2.8% DSC and 41.5%, 43.6%, and 43.1% ASD from theyoungest (32–36) to the oldest(<46)age group and reachesa DSC of 96.86, 96.913, and 97.17 for the cortical structures, 98.96,98.652, and 98.362 for the WM structures, and 92.18, 92.69, and 92.80 forthe subcortical structures across all age groups (youngest to oldest) on theT2w MRIs (Fig. 7, top). Here, VINNAalso reaches the lowest ASD (0.080 mm, 0.081 mm, and 0.081 mm for thecortical structures, 0.067 mm, 0.068 mm, and 0.066 mm for the WM structures,and 0.231 mm, 0.212 mm, and 0.212 mm for the subcortical structures). Theresults are significantly better compared to 3D nnUNet + exA(corrected$p<10^{-9}$).As seen by the increasing DSC and decreasing ASD, the younger age groups(<32−36)have proved to bemore challenging to segment. For VINNA, the performance decreases mostsignificantly for the subcortical structures (DSC by 1.82% and ASD by20.09%) and least on the cortex (0.35% DSC and 4.65% ASD). This trend isconsistent for the other two models. Assessment of qualitative differencesbetween the 3D nnUNet + exA and VINNA on a representative participantat 40 weeks of age (Fig. 10) showsslight over-segmentation of the cortex and loss of small WM strands with the3D nnUNet + exA (third row, second column, arrows). Overall, thesegmentation with VINNA (fourth row) appears less smoothed and closer to theground truth (second row).

#### 3.2.2 Comparison to iBEAT

InFigure 8, the deep-learning modelsare compared to the docker version of iBEAT on the T2w images at 0.5 mm.iBEAT is officially designed for ages 0–6 years and the dockerversion we used for processing returns three labels (WM, GM, CSF). Thedefinition of these labels is different from that of the dHCP-atlas (seeSection 2.3.3), so we map theground truth as well as the predictions from the deep-learning networks(nnUNet3D and VINNA) to be able to compare segmentation similarity. Asdescribed inSection 2.3, retrainingthe CNN part of iBEAT under same data and label definitions is not possible,as neither the source code nor the original training data is availableonline. Note, even though we do not need to interpolate, cross-protocolcomparisons include atlas differences and may introduce additional errorsdue to the mapping. While results should be interpreted with the caveat thatiBEAT uses a different atlas and training dataset than 3D nnUNet +exA and VINNA, the label harmonization allows an as-fair-as-possiblecomparison with this state-of-the-art method.

**Fig. 8.**
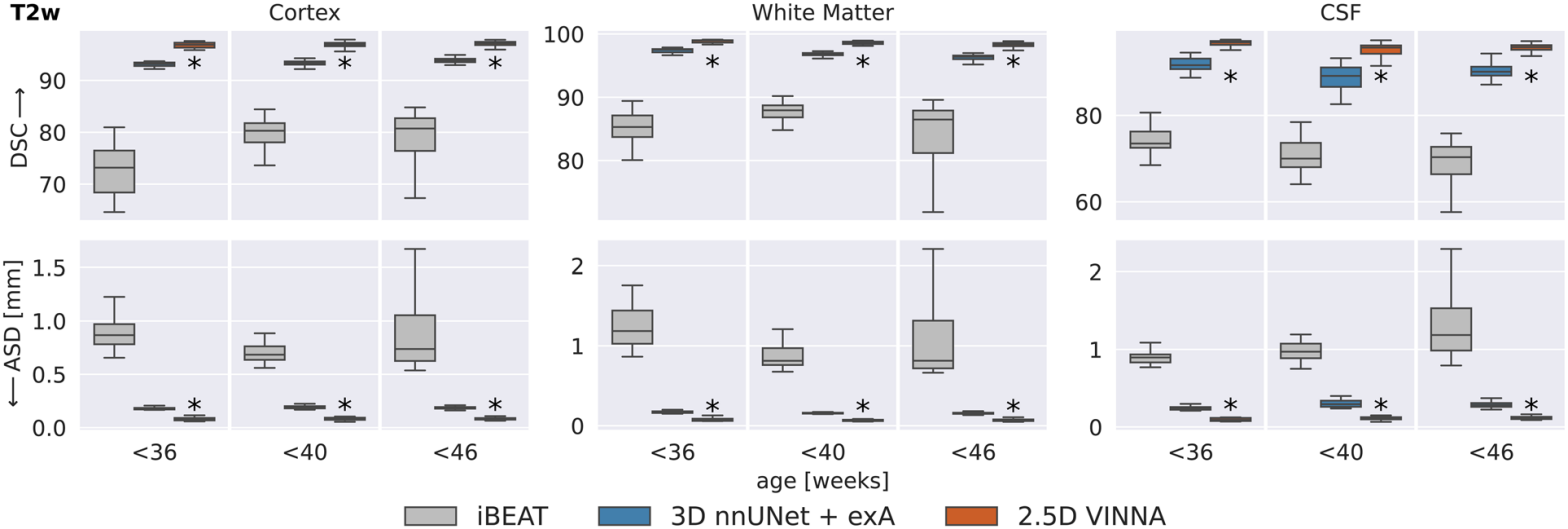
Deep-learning networks versus iBEAT: Similarity to the dHCP referenceis higher with VINNA (third box) and 3D nnUNet + exA (secondbox) than iBEAT (first box) with respect to dice similaritycoefficient (DSC, top) and average surface distance (ASD, bottom) onT2w MRIs at 0.5 mm across three age groups. Segmentation resultswith VINNA are significantly closer to dHCP (corrected$p<10^{-6}$,indicated with *) for CSF, GM, and WM. Note thatiBEAT’s structure definition is not identical to thedHCP-ALBERTs atlas and analysis is based on harmonized, mergedlabels.

With respect to the mapped dHCP-reference segmentation, DSC (top) and ASD(bottom) are lower for iBEAT (left box in each plot) compared to the thedeep-learning methods (3D nnUNet + exA, middle box, and VINNA, rightbox in each plot). Performance of iBEAT on the GM, WM, and CSF improves withage. GM and WM are closest to the reference at 36–40 weeks (DSC80.29/87.94 and ASD 0.6840.813 mm), while CSF peaks at 32–26 weeks(73.49 and 0.896 mm). The 3D nnUNet + exA and VINNA show a similartrend, but performance is more consistent. As mentioned before, thedifferences are not necessarily due to wrong predictions made by iBEAT.Looking at the qualitative comparison inFigure 10, differences appear small, with iBEAT (third row)missing a few WM strands (arrow) and slightly over-segmenting the cortexcompared to the mapped ground truth (second row).

#### 3.2.3 Comparison to infantFS

**Fig. 9.**
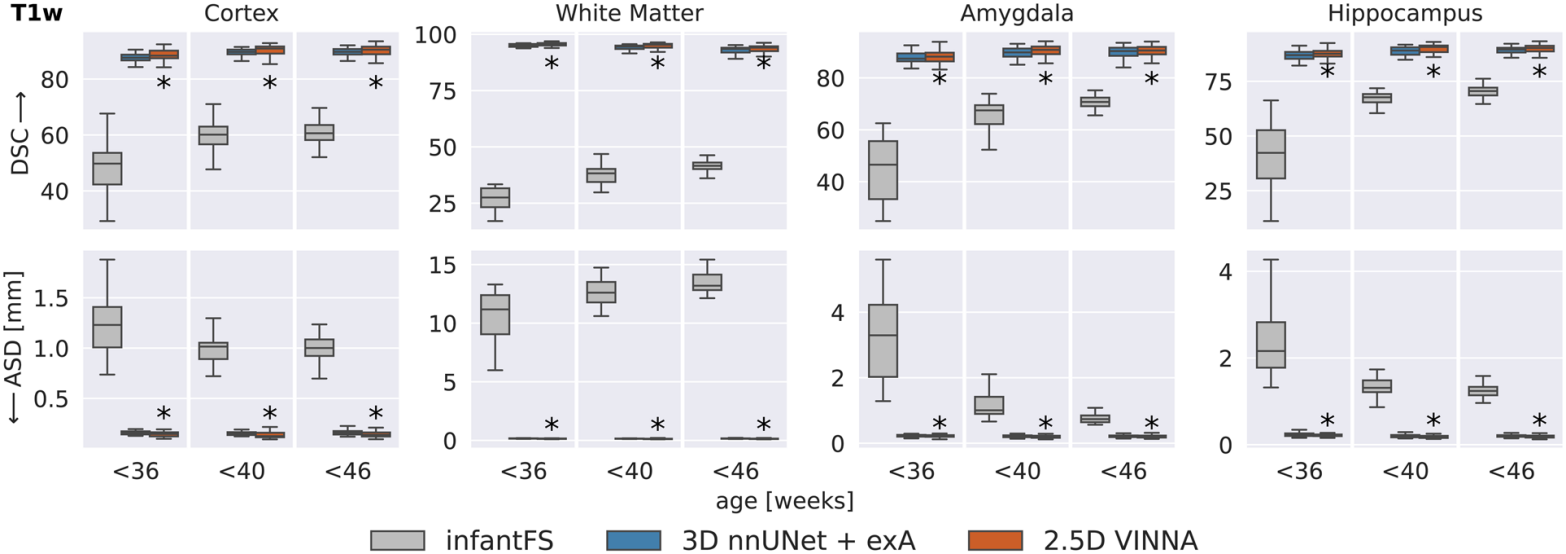
Deep-learning networks versus infantFS: VINNA with the 4-DOFtransform module (third box) and 3D nnUNet + exA (second box)are closer to the dHCP reference than infantFS (first box) on T1w at1.0 mm, the supported modality and resolution for infantFS. DiceSimilarity Coefficient (DSC, top) and average surface distance (ASD,bottom) significantly improve with VINNA (corrected$p<10^{-6}$,indicated with *) on the cortex, WM, hippocampus, andamygdala across all age groups. Note that definition of subcorticalstructures differs in infantFS and predictions are harmonized toallow comparison to the deep-learning models.

Figure 9shows performance comparisonbetween infantFS (left box) and the deep-learning methods, 3D nnUNet+ exA (middle box) and VINNA (left box), but in contrast to previousevaluations on T1w images at 1.0 mm, the operating resolution and modalityof infantFS. Note that the infantFS labels are also different from those ofdHCP and the ground truth labels must be mapped (seeSection 2.3.3for details). As for iBEAT, thecross-protocol comparison can put the traditional method at an unfairdisadvantage and results should be considered with this caveat.

Overall, the infantFS predictions differ strongly from the mapped dHCP groundtruth, specifically for the younger age ranges. The highest similarity isreached for the subcortical structures (amygdala and hippocampus) at40–46 weeks (DSC of 70.72/70.56 and ASD of 0.7361.23 mm,respectively). The cortex and WM reach a maximum DSC of 60.60/41.62 and ASDof 1.013.20 mm. Qualitative comparison (seeFig. 10, third row, left panel) shows difficulties with thecorrect location of the GM and WM border on the dHCP T1w MRI. Largerportions of the cortex are under-segmented, and strands of WM are lostcompared to the ground truth. The deep-learning methods reach a DSC above 80and an ASD below 0.5 mm for all structures and age groups. The methodclosest to the dHCP reference is again VINNA with the 4-DOF transform module(left box), followed by 3D nnUNet + exA (middle box). For a detailedcomparison between nnUNet and VINNA on T1w, seeFigure 7.

**Fig. 10.**
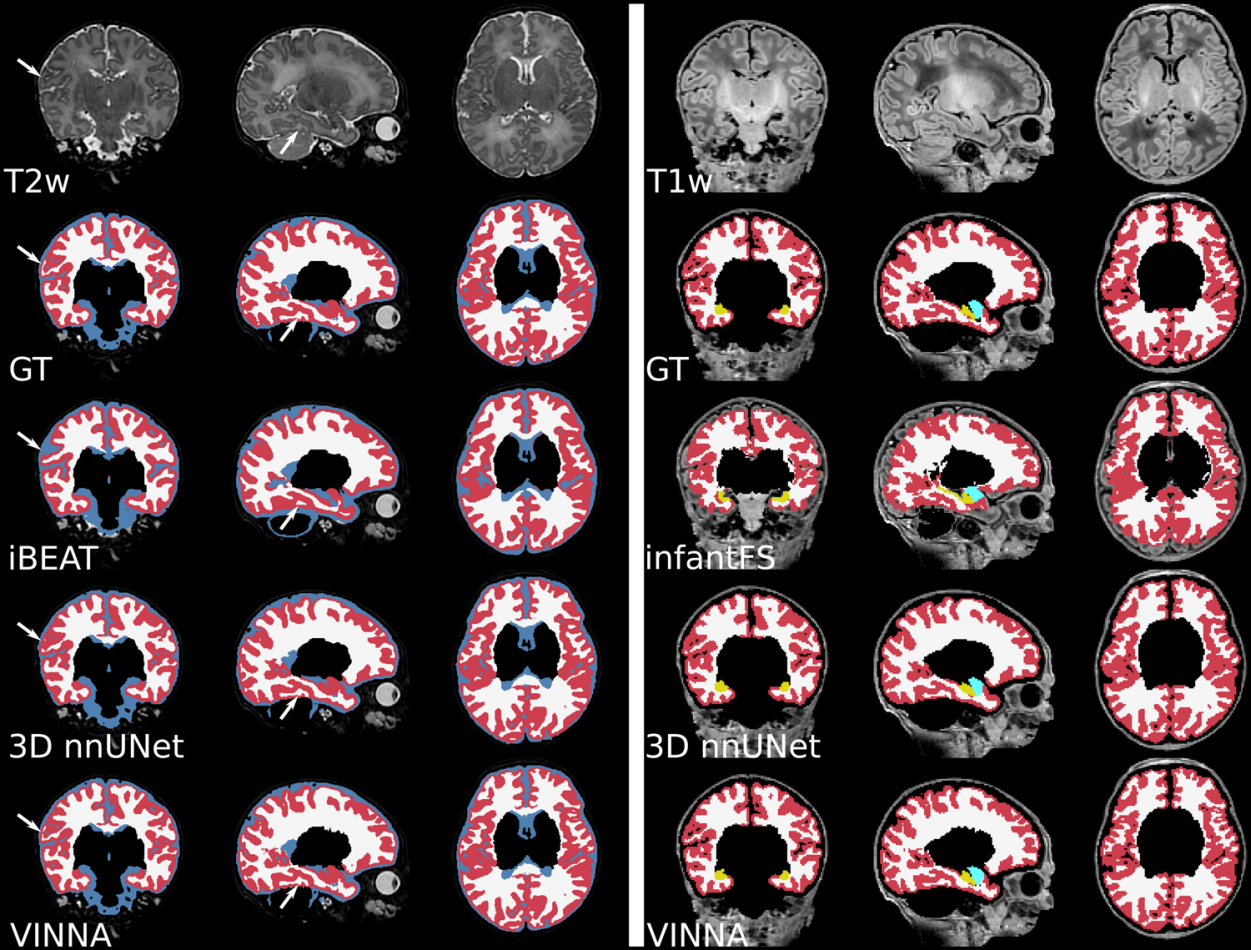
Qualitative T1w and T2w MRI segmentations on a representative scan at41 weeks. VINNA with the 4-DOF transform module (last row) capturesstructural details lost in other methods. Comparison of the mappedground truth (top) and segmentations from iBEAT (third row), 3DnnUNet + exA (fourth row) on a representativeparticipant’s T2w MRI (left). The right side shows theT1w-scan from the same participant at 1.0 mm with ground truth(top), infantFS (third row), and the deep-learning methods.

### 3.3 External validation on manual labels (M-CRIB)

To assess generalizability to a different dataset in our target age range(24–44 weeks) and to provide results with respect to a manual reference,we compare the segmentations produced by VINNA, 3D nnUNet + exA, and thedHCP-minimal-processing-pipeline to the 0.62 mm high-resolution T2w scansforming the M-CRIB atlas (Alexander et al.,2019). This dataset contains T2w MRIs from 10 participants andaccompanying label maps based on the Desikian-Killiany-Tourville (DKT) atlas(Klein & Tourville, 2012).Note that the labels are not identical to the dHCP-ALBERTs atlas. Hence, wecombine the cortical parcels to one label (cortex) for the segmentationcomparison and mask all but three structures (WM, hippocampus, and lateralventricles). As iBEAT does not differentiate between subcortical structures andGM or WM, nor CSF and ventricles, mapping of both, the ground truth andprediction, would be different compared to nnUNet, VINNA, and thedHCP-minimal-processing-pipeline. A fair comparison is therefore only possiblebetween the latter methods, and iBEAT is thus not included in the followingsection.

InFigure 11, the DSC (top) and ASD(bottom) are compared over four structures (from left to right: cortex, WM,hippocampus, and lateral-ventricles) for the deep-learning methods (3D nnUNet+ exA and VINNA) as well as the dHCP-minimal-processing-pipeline.Predictions for these methods are all based on the same label definition(dHCP-ALBERTs atlas (Gousias et al.,2012;Makropoulos et al., 2014)).As for the dHCP test set, VINNA outperforms the 3D versions of nnUNet across allfour structures with a DSC of 83.83 and an ASD of 0.387 mm on the cortex, 78.41and 4.268 mm on the WM, 61.73 and 3.682 mm on the hippocampus, and 84.62 and0.585 mm on the lateral ventricles. Compared to nnUNet, the performance improveson average by 3.36% for the DSC and 19.40% for the ASD. Furthermore, 3D nnUNet+ exA incorrectly flips left-right labels for five participants (P02-P04,P08-P09). We restore the lateralization in the presented results as DSC and ASDwould have otherwise been close to zero for half of the participants. On thehippocampus, ventricles, and WM performance of VINNA is similar to thedhcp-minimal-processing-pipeline, which is the best performing method.Specifically, predictions of the cortex are closer to the M-CRIB manual labelswith the dhcp-pipeline (DSC of 89.60 and ASD of 0.257 mm), which is likely dueto the reliance on surface models, while the trained deep-learning models seemto over-segment the cortex (indicated by the similarity of the ASD, but largerdifferences in the DSC). Note that VINNA performs slightly better on thehippocampus with an increase in DSC by 1.25% and ASD by 0.58%. Due to the lownumber of participants, significance tests are not applicable for theseexperiments.

**Fig. 11.**
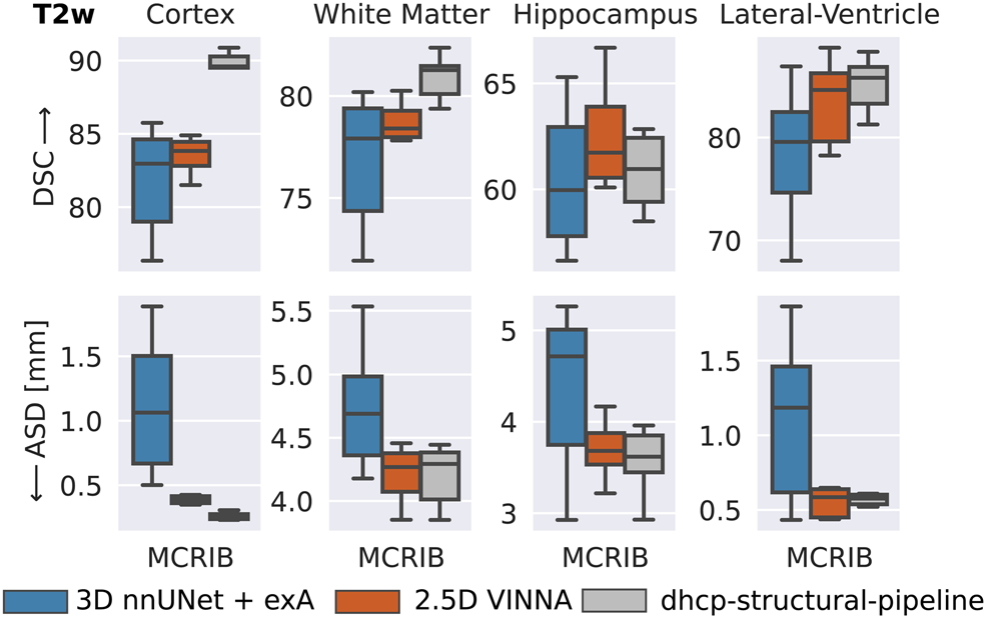
External validation of segmentation performance on the M-CRIB dataset.VINNA (second box) outperforms nnUNet3D (first box) on all fourstructures and the dHCP-strucutral-pipeline (third box) on thehippocampus. Dice Similarity Coefficient (DSC) and average surfacedistance (ASD) for cortex, WM, hippocampus, and lateral-ventricles arecalculated with respect to harmonized manual labels on 10 subjects fromM-CRIB. Note that atlas definition differs between ground truth andpredictions.

## 4 Discussion

In this paper, we present VINNA—a resolution-independent network fornative-resolution neonatal brain MRI segmentation. With VINNA and our novelnetwork-integrated 4-DOF transform module, we address two main difficultiesassociated with neonate segmentation: resolution non-uniformity across data cohortsand the extended range of head positions in infants.

In contrast to adults, newborn head positioning in the scanner varies significantlydue to imaging during sleep, smaller head sizes, and relevant necessarymodifications to scanner equipment, such as the padding of head coils. Additionally,while scans are commonly recorded at high resolutions, no uniform standard existsacross cohorts. The availability of newborn datasets is also scarcer than that ofadult subjects, and the existing collections to date are unlikely to represent thatwide diversity in resolutions and head positions.

The current state-of-the-art to address spatial variability such as head positions isdata augmentation, which applies randomly sampled scale, rotation, and translationtransformations in the native imaging space (i.e., externally to both intensity andlabel map). In VINNA, we introduce the 4-DOF transform module that can apply suchtransformations internally as part of the network. As the parameters to thetransformation are inputs to the network, they can be randomized during training,similarly to external data augmentation methods. Moving the augmentation operationinto the network, so it acts upon feature maps instead of inputs, marks a novelshift for data augmentation strategies. While we only implemented an augmentation of4 DOFs here, the concept may be generalized to 9 DOFs for 3D or even to warp fieldsas well as to other tasks such as classification, regression, etc.

We demonstrate that the new network-integrated 4-DOF transform with internalaugmentation outperforms state-of-the-art external augmentation approaches inCNN*, VINN, and nnUNet (Isensee et al.,2021) on the dHCP cohort. Across three different resolutions and twomodalities, our VINNA achieves the highest DSC (95.33 on average), as well as thelowest ASD (0.102 mm on average). Metric evaluation combined with qualitativeinspection indicates that the internal 4-DOF transform module in VINNA betterretains high-level details across all resolutions and age groups. It should be notedthat VINNA performs augmentation of the feature maps in the first-scale transition.The first (pre-IDB) and last (post-CDB) blocks, therefore, act independent of anyinterpolation and promote feature detection at an unaltered scale. While theablative results inFigure 5indicate betterperformance than with external augmentation, generalization beyond the scalesencountered during training is not assured in these two blocks. Due to theirsimplicity, the initial, low-level features are, however, often empiricallyresolution-independent by nature (e.g., Line-Detector).

To better explain the factors and mechanisms driving the performance improvements ofVINNA, we review the observation from FastSurferVINN (Henschel et al., 2022) that motivated the extensionpresented here: in one-to-one comparisons, external augmentation reduces thesegmentation performance on sub-millimeter MRIs. While—at firstsight—the addition of data augmentation reducing performance seemscontradictory, the one-to-one comparison of VINNA and VINNA + exA (seeFig. 5, the only difference is added externalaugmentation) robustly confirms the observation and extends it from just scaling torigid transforms.

The positive effect of data augmentation is usually associated with an expansion ofthe input dataset through equivariant operations. Implementing operations for imageand label pairs that are truly equivariant can be difficult. We believe that theloss of information due to image interpolation (lossy interpolation of the label mapand image) is larger than previously believed. Internal augmentation, for the firsttime, offers an alternative approach with interpolation of continuous values inmultiple feature maps, reducing the information loss.

Furthermore, the 4-DOF transform module together with the internal augmentationregularizes the latent space of the network, because it imposes an additionalconstraint: spatial consistency of the feature maps. Compared to equivalent CNNarchitectures, VINNA (and VINN) also benefit from a reduced capacity requirement tocapture a large range of resolutions.

Our comparison to nnUNet highlights that 2D approaches lack contextual information,and fail to provide reliable predictions for whole-brain segmentation. Thecompromise between mid-range and long-range context in the 2.5D VINNA recoversstructural information better and achieves higher segmentation performance acrossall age groups and structures—even compared to 3D methods. As full-view 3Dnetworks are currently not applicable for high-resolution MRI segmentation due tomemory requirements, nnUNet and other 3D networks rely on patch-based processing. Inthis case, the increased 3D context comes at the cost of limited long-rangeinformation and features a smaller field of view, potentially explaining theobserved reduction in accuracy compared to 2.5D networks. This finding is in linewith previous investigations which found limited performance differences between2.5D and 3D approaches, even after extensive optimization of the 3D networkarchitectures (Roy et al., 2022).

On the dHCP cohort, VINNA and its 4-DOF transform module also emulates the segmentedstructures better than traditional state-of-the-art infant pipelines, namelyinfantFS (Zöllei et al., 2020) andiBEAT (Wang et al., 2023). Notably, infantFSrelies on traditional atlas-based segmentation while iBEAT uses a combination ofdeep-learning and traditional tools with a number of CNNs trained on defined targetage ranges. While the re-trained networks (nnUNet + exA and VINNA) reachbetter results with respect to DSC and ASD, it should be noted that both, infantFSand iBEAT, differ significantly with respect to the returned number and definitionof segmented regions. The necessary mapping between the segmentations is bound tointroduce a bias, which can not be easily assessed. Additionally, both pipelinescater to a slightly different, larger age range (0–2 years for infantFS and0–6 years for iBEAT). Consequently, predictions from both methods of thecortex and WM improve for participants closer to the officially supported age range(>40 weeks). Qualitative assessment also shows good performance for the oldernewborns in iBEAT. infantFS unfortunately fails to correctly capture the cortex andWM on the majority of participants.

The original iBEAT v2.0 paper (Wang et al.,2023) also evaluates performance on the dHCP data and reports a DSC of0.9 for the WM and 0.85 for the GM. Our results are in concordance with thisassessment for the >40 week-old participants (DSC of 0.83 on the GM and 0.88on the WM). The authors do not provide information on their label harmonization,therefore we cannot infer their reference standard. In contrast to the docker v2.0version, the cloud version of iBEAT (not available for local data processing) doesprovide cortical parcellations. Extracting the cortex from the GM label (acombination of both cortical and subcortical GM in the docker version) allows directcomparison to the dHCP solution after merging its cortical structures, possiblyexplaining performance differences. In summary, iBEAT seems to work well on thesupported age range while infantFS is less precise on the dHCP population. Otherfeatures included in the pipelines, such as surface generations, are an advantagecompared to the proposed VINNA and can help to refine the segmentation of convolutedstructures such as the cortex (Fischl, 2012).For our target domain in this paper, however, the VINNA architecture appears toemulate the investigated tissue classes more precisely.

Due to the limited extrapolation capabilities of neural networks, generalizabilitybeyond the training set distribution is, however, uncertain. While the 4-DOFtransform module in VINNA serves as a diversification of the training distributionwith respect to spatial orientations and image resolution, the base cohort is stillonly a representation of the dHCP population, that is, all scans encountered duringtraining represent newborns between 24–44 weeks post-menstrual age from acontrol cohort acquired on the same 3 T Phillips scanner. Therefore, dedicatedexperimental validation is required to confirm the models’ effectivenessunder differing conditions. As for all automated methods, manual quality checks ofthe predictions are recommended. While VINNA does perform well on M-CRIB, whichcovers the same age range as the dHCP, generalization to other cohorts is notnecessarily guaranteed. Specifically, the T1w image intensities in dHCP appearsignificantly different from other cohorts which might also explain why infantFSperforms poorly on the testing set. The T2w MRIs in dHCP are, on average, of betterquality and the dhcp-minimal-processing-pipeline builds the ground truthsegmentations based on it (Makropoulos et al.,2018). Additionally, in the early weeks of life, tissue contrast ishigher in T2w recordings as the brain is not fully matured and myelination is stillongoing (Dubois et al., 2021;Miller et al., 2012). Structural details and specificallytissue boundaries might be missing, blurred, or ambiguous in T1w MRI. Hence, theimaging data may lack sufficient information to allow correct delineation of the(sub-)cortical structures. This may also explain why the deep-learning networks(i.e., nnUNet, CNN*, VINN), and VINNA are not able to emulate the groundtruth on the T1w MRI as closely as on the T2w images. Including a SynthSeg-likeintensity augmentation can potentially aid generalization across a wider age range.The method has previously been used in adults to generate contrast-agnostic networksthat are able to segment both T1- and T2-weighted images (Billot et al., 2020;Iglesias et al., 2021). Due to the strong contrast changes in the earlydevelopmental years, implementing such a generative model may be an interestingdirection for future work. However, as mentioned in the introduction, adaptation tothe newborn cohort (Shang et al., 2022)showed that the synthetic images still differ considerably from real data leading toperformance reduction compared to age-specific models trained on real images.Successful adaptation requires a strategy to close this performance gap.

Better accessibility of newborn datasets would allow diversification of the trainingsets and subsequently a better representation of the newborn MRI distribution withrespect to both, T1w and T2w modalities. It has been shown that an increase in thetraining corpus alone is extremely effective to boost performance (Henschel et al., 2022;Sun et al., 2017). Age-specific models, such as our VINNA oriBEAT’’s CNNs, are another way to reduce variations and therefore,segregate the problem (i.e., less variations within one age group). However, thelimited data availability and non-uniform segmentation labels still pose significantbarriers. Models for more specific segmentations than just CSF, WM, and GM arecurrently not trainable in a supervised fashion due to missing ground truth. Inaddition, definition of these three structures alone already varies across differentatlases and tools, which makes fair method comparisons challenging. Neither manuallabels nor automated segmentation tools exist for a unified segmentation definitionacross different resolutions, modalities, and age ranges. The M-CRIB atlas (Alexander et al., 2019), an infant-specificversion of the Desikan-Killiany-Tourville-Atlas (Klein & Tourville, 2012) that is commonly used in adults,provides a first step towards this goal. A consistent structure definition acrossdifferent stages of life is especially important in the context of longitudinalstudies, as segmentation with age-dependent models can induce biases and reduceanatomical consistency (Reuter et al., 2012).How to solve this conundrum is an open question for the future. Several NIH- andinternationally-funded initiatives have recently been dedicated to acquire data fromnewborns (Makropoulos et al., 2018), infants,and pediatric age ranges (Howell et al.,2019;Jernigan et al., 2016;Volkow et al., 2021) as well as adolescence(Karcher & Barch, 2021). Due tothe easy integration of varying data resolutions and accommodation for head positionvariations between infants, toddlers, and adults, our VINNA architecture might proveto be useful in this area once the data and label availability problem isresolved.

Overall, with VINNA, we provide a fast and accurate method for high-resolutionsubcortical structure segmentation, cortical and WM parcellation of neonatal T1w andT2w MRI which generalizes well across the dHCP cohort. The presented 4-DOF transformmodule is also easy to integrate into other network architectures and might proveuseful in different areas dealing with strong orientation variations. Theapplication to neonates will be made available under VINNA4neonates as an opensource package.^8^Adaptation of theinfantFS surface pipeline to the VINNA4neonates predictions, similar to the approachtaken in FastSurfer (Henschel et al., 2020)for adults, is an exciting direction for future work.

## Figures and Tables

**Appendix Fig. A1.**
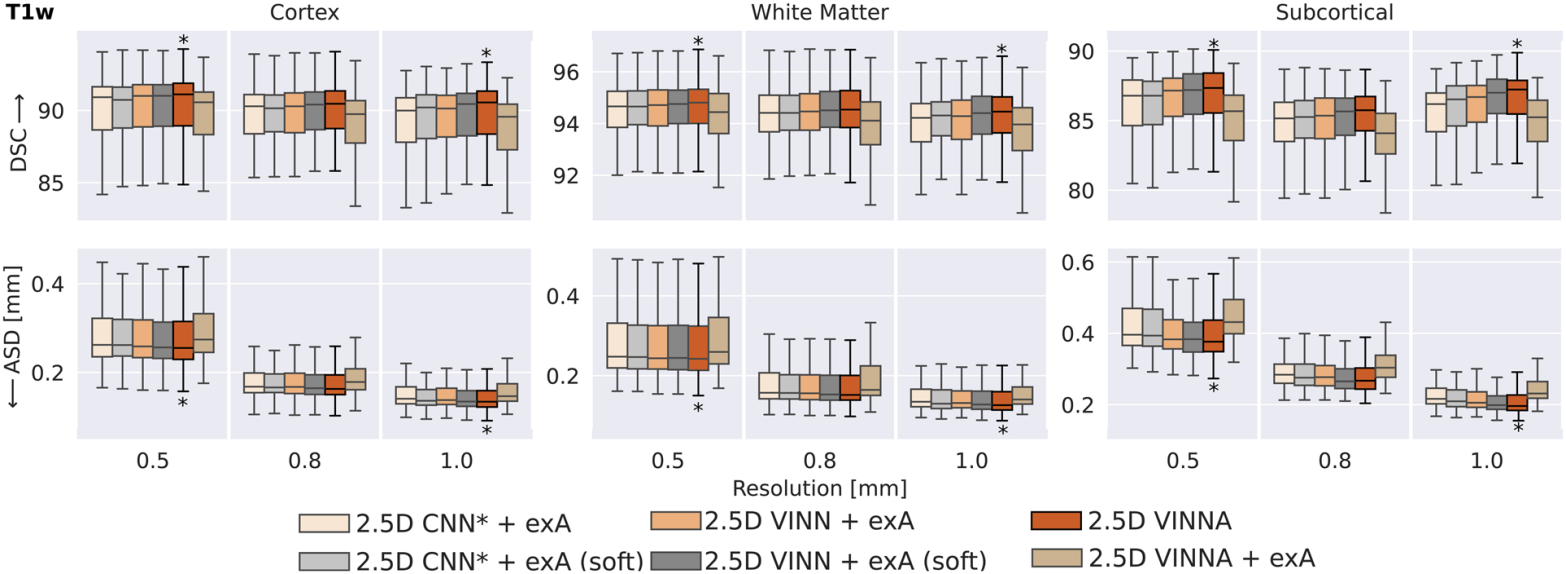
Comparison of approaches for external and internal spatial augmentationon T1w images: Our VINNA method—with the 4-DOF transformmodule—(fifth box in group) outperforms both state-of-the-artapproaches with external Augmentation (CNN* + exA,CNN* + exA (soft), VINN + exA, and VINN +exA (soft); first to fourth box) in Dice Similarity Coefficient (DSC,upper row) and average surface distance (ASD, lower row). Thisperformance advantage is significant on 0.5 mm and 1.0 mm (correctedp<0.05,indicated with *) and consistent across structures. CombiningVINNA with external augmentation (VINNA + exA, last box) reducesperformance. exA: external Augmentation. soft: linear interpolation ofone-hot encoded feature maps.

**Appendix Table A.**
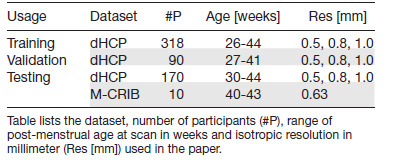
Summary of datasets used for training, validation, and testing.

**Appendix Table B.**
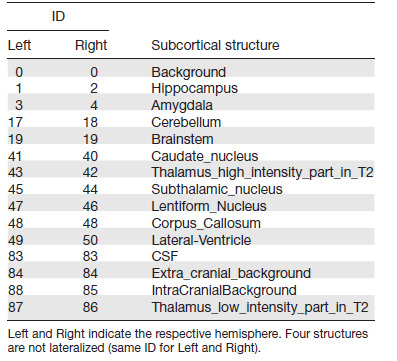
Subcortical segmentations of the dHCP-ALBERTs atlas and matchingidentifier (ID) equaling the number returned by VINNA for thestructure.

**Appendix Table C.**
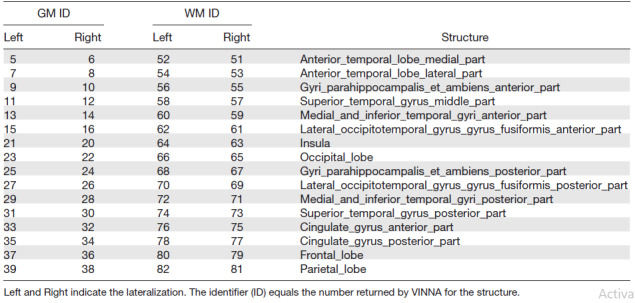
Segmentation parcels of the dHCP-ALBERTs atlas for gray matter (GM)and white matter (WM).

**Appendix Table D.**
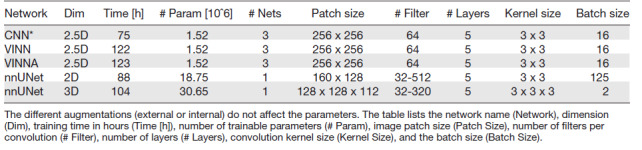
Summary of training parameters for CNN*, VINN, VINNA, nnUNet2D and nnUNet 3D.

## Data Availability

All MRI datasets used within this article are publicly available, and the open sourcerepositories are cited within the article (Section2.1). The source code of VINNA4neonates will be made publicly availableon Github (https://github.com/deep-mi/VINNA4neonates) upon acceptance.

## Footnotes

1 github.com/Deep-MI/VINNA4neonatesupon publication

2
https://github.com/iBEAT-V2/iBEAT-V2.0-Docker

3
https://surfer.nmr.mgh.harvard.edu/fswiki/infantFS

4
https://data.developingconnectome.org/

5
https://ibeat.wildapricot.org/

6
https://github.com/iBEAT-V2/iBEAT-V2.0-Docker

7 To reduce redundancy, we relegate that analysis to the Appendix (Section A.1).

8 on our github pages.

## Appendix

### A.1 Ablation on T1w

AppendixFigure A1shows DSC (top) and ASD(bottom) for the ablative evaluation external augmentation (exA), optionallywith linear interpolation of the one-hot encoded feature maps (exA (soft))instead of hard label resampling, versus the internal 4-DOF transform modul(VINNA) on the T1w. CNN* + exA (first box) and + exA (soft)(second box) reach the weakest results, followed by VINN + exA (thirdbox) and VINN + exA (soft) (fourth box). Finally VINNA (fifth box)reaches the best result. The performance improves the most on the subcorticalstructures (2.44% ASD and 0.4% DSC when switching from CNN* to VINN,5.91% ASD and 0.85% ASD when switching to VINNA). Performance improvements withthe 4-DOF transform module in VINNA, is significant for 0.5 mm and 1.0 mm(correctedp<0.05) andresults in an average final DSC of 90.95, 90.03 and 90.36 and ASD of 0.295 mm,0.2 mm, 0.2 mm for the 0.5 mm. 0.8 mm, and 1.0 mm resolution, respectively.Re-addition of exA to VINNA (sixth box) reduces performance to a similar levelas CNN* + exA.

Overall, all models perform better on T2w (Fig.5) rather than T1w MRIs (Appendix Fig.A1) across all structures. When segmenting a T2w MRI of the sameindividual, peformance improves for all networks, on average, by 5.7, 3.63 and5.09 DSC points for the cortex, WM, and subcortical structures, respectively.The ASD difference between T1w and T2w applications nearly doubles for thehighest resolution (0.5 mm) with an average of 0.18 mm compared to both lowerresolutions (ASD of 0.9 mm for 1.0 and 0.8 mm) for the three structure averages.The DSC point improvement for the T2w images is similar across all resolutions.The trend is consistent for both networks (CNN* and VINN) andaugmentation schemes (external versus internal).
